# Medical Tourism and the Best Interests of the Critically ill Child in the Era of Healthcare Globalisation

**DOI:** 10.1093/medlaw/fwaa029

**Published:** 2020-10-08

**Authors:** Neera Bhatia, Giles Birchley

**Affiliations:** 1 Associate Professor, Deakin University, School of Law, Melbourne, Australia; 2 Research fellow, University of Bristol, Centre for Ethics in Medicine, UK

**Keywords:** Best Interests, Disputes, Globalisation, Innovation, Medical Tourism, Social Media

## Abstract

In this article, we examine emerging challenges to medical law arising from healthcare globalisation concerning disputes between parents and healthcare professionals in the care and treatment of critically ill children. We explore a series of issues emerging in English case law concerning children’s medical treatment that are signs of increasing globalisation. We argue that these interrelated issues present distinct challenges to healthcare economics, clinical practice, and the operation of the law. First, social media leverages the emotive aspects of cases; secondly, the Internet provides unfiltered information about novel treatments and access to crowdfunding to pay for them. Finally, the removal of barriers to global trade and travel allows child medical tourism to emerge as the nexus of these issues. These aspects of globalisation have implications for medicine and the law, yet child medical tourism has been little examined. We argue that it affects a range of interests, including children’s rights, parents’ rights as consumers, and the interests of society in communalised healthcare. Identifying putative solutions and a research agenda around these issues is important. While cases involving critically ill children are complex and emotionally fraught, the interconnectedness of these issues requires the law to engage and respond coherently to the impacts of healthcare globalisation.

## I. INTRODUCTION

Decision-making for critically ill children can be complex, protracted, and distressing for the child, the parents, and the treating healthcare professionals, and can engender strong sentiments in wider society.^1^ There is increasingly an expectation that new technologies and medical science will extend the lives of patients, including children with life-limiting conditions.^2^ In tandem, long-term policy trends have driven a consumerist approach to healthcare and asserted greater patient empowerment.^3^ Parents, too, may be becoming increasingly assertive consumers on behalf of their children in their interactions with healthcare providers.^4^ These developments have taken place against the background of growing healthcare globalisation that presents challenges to both the delivery of children’s medicine and, increasingly, the law.

In English law, medical treatment decisions for critically ill children are made based on their best interests. Where the child is unable to participate, these are usually agreed upon through a ‘shared decision-making’ process^5^ involving the treating healthcare team and parents. Sometimes, however, parents and healthcare professionals disagree about which treatment, if any, is in the best interests of a critically ill child. The most contentious disagreements can occur when limits to life-sustaining treatment are at issue. Parents of a critically ill child may request the continuation of life-sustaining medical treatment, sometimes through the use of novel or innovative treatments, against the clinical opinion that further treatment is in the child’s best interests.^6^ In some cases, this opinion is due to doubtful effectiveness and/or uncertain risks of novel therapies.^7^ In others, the treatment requested may be relatively established yet not considered appropriate by the treating team.^8^ Alternatively, parents may refuse to consent to a specific medical treatment for their child, contrary to medical advice that the treatment is in their child’s best interests.^9^ In each of these types of cases, when disputes between parents and healthcare professionals about a child’s medical treatment reach an impasse, court intervention is required to determine what is in the best interests of the child.^10^

Like many other areas of health law,^11^ the challenges of globalisation—by which is meant a range of interrelated processes that break down the barriers caused by distance to the flow of information, people, and goods—are gradually becoming evident in the law surrounding children’s medical (non) treatment. The purpose of this article is to examine these particular challenges by exploring disputes concerning children’s medical treatment in English law. Through an examination of these cases, we illuminate and discuss three interrelated effects of globalisation. First, the Internet both allows the transfer of information and facilitates social media’s harvesting of data on ‘emotional states and triggers’.^12^ The first-generation, ‘information’ Internet (sometimes known as Web 1.0) allows parents to access a global knowledge base, where information about numerous treatments is available, largely unfiltered by caveats concerning likely efficacy or appropriateness, increasing the pressure for innovative or unusual treatments to be adopted. The second-generation Internet of social media (sometimes known as Web 2.0) allows parents to establish—and social media algorithms to exponentially expand—a social network of emotionally involved third parties. While this is a potentially valuable counter for the isolating effects of the hospital environment, the innate bias of social media towards emotional expression allows parents not only to publicise disagreements on a global scale but also to be swept up in the wave of emotional outrage this triggers in supporters. The establishment of an emotionally motivated, globalised network polarises disputes and provides a potentially large pool of ‘crowdsourced’ funding. The nexus of readily available information on novel treatments, networks of emotional connection, and private funding is, lastly, an increasing influence for child medical tourism. In a global healthcare market, most treatments will be readily available, in part due to national differences in regulatory norms and cultural attitudes towards children. Ready access increases pressures on, and provides opportunities for, parents to participate in these markets. Each of these effects of globalisation presents distinct challenges to medical law which we discuss in later sections. First, however, we provide some background to the law as it pertains to medical treatment and children. This is followed by a discussion about globalisation in the healthcare context.

## II. CHILDREN’S MEDICAL TREATMENT AND THE LAW

With the USA the only current non-ratifying country, the 1989 UN Convention on the Rights of the Child (UNCRC) is the international legal norm in decision-making for children. The convention asserts that in any decision affecting a child, the best interests of the child are a primary consideration.^13^ While the convention is not incorporated into English law, the Children Act 1989 (CA89) follows similar principles to the UNCRC. The Act, which brings together a complex array of private and public law relating to children within a single statute, states that the child's welfare is the paramount consideration in any decision.^14^ Like the UNCRC,^15^ CA89 affirms the central position of parents as decision-makers for children, while construing the role of parents in terms of responsibilities towards their child rather than rights over them.^16^ Inasmuch as parents have rights, these are rights to decide according to the child's welfare.^17^ CA89 has also been argued to implicitly favour partnership between parents and other agencies.^18^

This partnership approach is overtly favoured in English common law, which argues that neither parents nor medical practitioners can lawfully make decisions for a child in isolation.^19^ Only when and if this shared decision-making process breaks down, should the courts be approached for a definitive decision.^20^ Since 1981,^21^ the courts have regularly been required to intervene in disagreements about children's medical treatment.^22^ Common law in this area is centrally premised on the principle of ‘best interests’,^23^ a principle criticised by some academic scholars as ‘an empty mantra’^24^ that allows discretionary decision-making.^25^ This approach may arise from a desire to avoid the dangers of making the parameters of intervention unduly narrow, a clear motivation in the Law Commission Report that preceded CA89,^26^ and the lack of specificity arguably necessitated in a pluralistic liberal state, given such a state should be wary of prescribing a particular view of the good life.^27^ In the case of incapacitated adults, the space left by the lack of specificity of the ‘best interests’ principle has increasingly been filled by accounts that seek to empower patients.^28^ Empowerment narratives are also commonplace in academic considerations of the parental role in children’s medical decision-making,^29^ although thus far resisted by the courts.^30^ Recourse to the courts may be rising—of the 46 children whose medical treatment was considered in published court reports between 2009 and 2019, 31 featured in cases taking place in the last 5 years. Yet, it is not simply that medical decision-making is increasingly litigious, but that the possibilities through which a disputed medical decision can be challenged are increasingly affected by the advance of globalisation. As we have noted, globalisation appears in cases of disputed medical treatment in three ways. First, the Internet brings unmediated access to information about novel or innovative treatments from global providers. Secondly, social media brings opportunities for global publicity, drives emotional connection, and potentially opens sources of funding. Thirdly, at the nexus of these trends, the global market is accessed in child health tourism.

## III. HEALTHCARE GLOBALISATION

In a classic definition, globalisation is the ‘spacio-temporal processes of change which underpin a transformation in the organisation of human affairs by linking together and expanding human activity across regions and continents’.^31^ While there are several recognised accounts of globalisation,^32^ the concept of globalisation has been argued to break down into several basic parts.^33^ In this reading, it comprises a series of long-term,^34^ interconnected, processes that cause space and distance to be increasingly immaterial to a range of social activities, connected events, and decisions. This results in the acceleration of the flow of information, goods, events, and people.

Globalisation is a multi-factorial economic, political, technological, and social phenomenon, with current drivers ranging from the re-organisation of international institutions according to neoliberal principles, the deregulation of national economies along laissez-faire economic lines, the rise of the Internet and development of social media, low-cost international travel, and the growth—and increasing power—of supranational social networks, non-governmental organisations, and multinational corporations.^35^ While globalisation has simultaneously been viewed as an opportunity and a challenge in healthcare,^36^ it is important to acknowledge that, for citizens of poorer countries, many of the putative benefits of healthcare globalisation have been dramatically one-sided.^37^ Internationalised business interests have long exploited weaknesses in national regulation to further their interests.^38^ It is true that the ease with which goods and services can move across international borders has led to some opportunities within low- and middle -income countries, whose governments sometimes actively foster a medical tourism industry as a means for economic growth.^39^ Yet, inequalities in healthcare access in such countries are worsened by both the insuperable economic and political barriers to accessing healthcare from,^40^ and the flight of skilled healthcare professionals to,^41^ wealthier jurisdictions. These inequalities may be made ever more acute by sharp disparities in facilities available for international health tourists and local populations,^42^ as well as the related reduction in the availability of health resources locally.^43^

Because of these deleterious effects on the most vulnerable, globalisation has since the late 1990s faced (paradoxically, often globalised) opposition from environmental groups and anti-poverty campaigners.^44^ Yet since the global financial crisis of 2008 globalisation has entered a new phase, facing a backlash from a number of populist, often authoritarian, political movements in wealthier nations. In the face of public concern in these nations over falling living standards and eroded social welfare, these movements have, sometimes successfully, and often capitalising on social media, employed emotional rhetoric asserting national identity and economic protectionism.^45^ Inherently opposed to a borderless world, these movements nevertheless appear dependent on, and inseparable from, the process of globalisation, and are thus potentially explicable in dialectic terms. Moreover, while globalisation has always involved the exchange of ideas, and the earlier (pre-global financial crisis) stage of globalisation was most associated with the expansion of trade, the current phase, fuelled by the rise of social media, has seen the interconnection of networks of actors based on their emotional sentiments^46^ become more dominant. Healthcare has not been immune to these changing winds.

While globalisation affects many healthcare variables in wealthy countries, including the cost of medicines and the makeup of the healthcare workforce, one of the most visible putative benefits for patients has been in opportunities for medical tourism. Medical tourists in wealthy countries apparently benefit both from increased access, choice, and reduced costs of treatment. Corporate actors, as well as individuals, have access to these benefits. In the USA, the latter factor has led to off-shoring of healthcare by medical insurers, and in the UK, rather more tentatively, the National Health Service (NHS) has followed a similar strategy to reduce patient waiting times.^47^ Surveys and first-hand accounts of patients indicate high levels of satisfaction with the quality—and reduced cost—of the care they receive.^48^ Nevertheless, these benefits may be questioned. High-quality evidence to inform consumer choices is limited, and it appears the patients’ choices of international providers are heavily influenced by personal testimonies within peer networks.^49^ While satisfaction is seemingly high, there is a paucity of high-quality studies to demonstrate this,^50^ with few studies systematically following up patients in the short or long term.^51^ Indeed, even if we disregard these caveats, the putative benefits of globalisation of healthcare may be more questionable. Healthcare practitioners may be affected by undercutting skills, standards, and prices in market-driven healthcare systems.^52^ Patients may find it difficult to seek legal redress from a practitioner in another jurisdiction should any aspect of treatment fall below the expected standard.^53^ Further, patients may also potentially face obstacles accessing remedial treatment in their country of origin, with some practitioners reporting concerns about their precarious legal position should they provide such treatment.^54^ Public healthcare systems in wealthier countries also face a constellation of challenges. Communalised public healthcare systems face potential costs when treatment initiated privately abroad requires follow-on treatments, or if they have to foot costs of remedial healthcare when things go wrong.^55^ Public health may directly be affected by the transmission of infectious diseases by health tourists.^56^ Nor is healthcare immune to recent trends linked to social media technologies, towards emotional connection in globalisation. These can arguably transform entire healthcare systems into totemic markers of identity—witness the focus on claims about the NHS in the UK’s Brexit referendum. Moreover, as we have seen in populist commentary around^57^—and some political responses to^58^—the global COVID-19 pandemic, health policy has potential to follow—and perhaps reinforce—trends already apparent in health consumerism in conceptualising health as a matter for autonomous (in this case, political) choice rather than as a physical need. Most significantly for our purposes, we have seen that the ready connection to the emotions of the global public can play a direct part in exacerbating existing disputes between the families of critically ill patients and healthcare providers.

The impact of globalisation on healthcare law has been considered in the context of human rights,^59^ global justice,^60^ and political economy.^61^ While we share many of the concerns commentators attach to the economic and political impacts of globalisation, this article focuses specifically on highlighting the effects of globalisation as it pertains to children’s medical treatment in English legal cases. Of these effects, the advent of child medical tourism, like its adult counterpart, is the most readily identifiable feature of globalisation at a case level. Yet, while a clear—although little studied^62^—area of concern, other push and pull factors within globalisation act on child medical tourism. These too have emerged as a feature of interest in cases of disputes about children’s best interests in medical treatment and must be examined to accurately understand the current context, and ultimately to address current problems. The role of social media in both opening up disputes to the sentiments and agendas of national and international third parties, as well as that of the Internet at large in the more basic process of facilitating information about novel and innovative treatments and their providers in the global marketplace is essential to the emerging issue of child medical tourism. Increasing interconnection facilitates the ready movement of information (in the form of formal or informal advertisement of services), emotional connection (including narratives of identity and belonging seen in the ascendant political movements), funding (in some cases), payments, parents, and (child) patients to destination countries where healthcare services are located.

Viewing these challenges through the lens of globalisation may appear excessively abstract. It may appear obvious that social media allows third parties to develop an emotional involvement in distant events or that the Internet allows a wider audience to seek and access novel treatments thus fuelling demand for medical tourism. If these connections appear trite, we suggest that this is so due to their familiarity, not their unimportance. These are the tangible elements of globalisation as it exists in our everyday lives. To focus on these challenges as components of globalisation is necessary if we are to offer both a coherent explanation of, and effective answers to, developments in this area of law. We shall now examine each of these key issues as they arise in disputes between parents and healthcare practitioners about the care and treatment of critically ill children.

## IV. ASPECTS OF GLOBALISATION AS EMERGING CHALLENGES WITHIN CHILDREN’S HEALTHCARE DECISION-MAKING

There are three aspects of globalisation that present challenges to medical and legal decision-making that are reflected in recent case law. Before we begin our discussion, two observations are necessary. First, while we choose to consider these challenges in a stepwise way, it is clear that these challenges did not necessarily emerge as a connected phenomenon, and it is only in the fullness of time that they have become interconnected in the way we suggest in our discussion. Secondly, these challenges have emerged gradually in case law, appearing in early cases as either minor or uncomplicated (if novel) aspects. Yet, each is now increasingly recognisable both as a challenge in its own right, and as a conduit of globalisation. The first of these challenges is the use of the Internet and social media by parents. The emergence of instant global communications is a central driver of the current phase of globalisation, with effects including the rise in globally orientated social movements.^63^ In children’s healthcare, social media have emerged as a means of publicity and emotional connection with a global public, and in some cases as a way to raise funds and embark on campaigns with a global reach. These effects are already causing repercussions in healthcare decision-making and family law.

### A. The Internet, Social Media, and Developments in Family Law

In *Roberts*,^64^ a seven-year-old boy, Neon Roberts, required post-operative chemotherapy and radiotherapy following surgery to treat a malignant brain tumour. The recommended treatments had an approximately 80–86% chance of success. Although his father (and children’s guardian) agreed to the treatment, his mother sought alternative treatments that she considered would prevent any long-term side effects of the proposed treatment. To prevent the cancer spreading, treatment was required immediately. However, Neon’s mother went missing, taking her son with her. They were located following a court order, and Neon was briefly placed in foster care and subsequently into the care of his father. The NHS trust applied for court declarations concerning Neon’s medical treatment. During the hearing, a magnetic resonance imaging (MRI) scan revealed another tumour requiring urgent surgery that his mother also opposed. The court determined it was in Neon’s best interests that the surgery took place. The case was sensationalised in the print media, largely unsympathetic to his mother’s objection to conventional cancer treatment and request for alternative treatments.^65^ Nevertheless, a blog was published^66^ detailing information, links to legislation, and reasons why Neon should receive alternative treatments. The blog included an open letter to the then British Prime Minister^67^ requesting authority for Neon to be taken abroad to receive alternative therapies. It also included a link to a donation page to raise funds for Neon to be taken abroad for treatment.^68^

There were echoes of these features in subsequent cases. Two years later, in *King*,^69^ the parents of five-year-old Ashya King sought access to proton beam therapy, one of the treatments requested in *Roberts*. Ashya’s parents considered this therapy to be a less damaging type of radiotherapy to treat his brain tumour. Proton beam therapy was not available in the UK. Having been advised by his doctors that the therapy would provide no additional benefit, and under the belief that the hospital would seek a child protection order to prevent them travelling, the family removed Ashya from the hospital and travelled to Spain. This precipitated the issuing of a European Arrest Warrant and sparked an international manhunt. His parents took to social media to make their case, and it was rapidly publicised in print and broadcast media. His parents were found, arrested, and separated from Ashya by the Spanish police. The media were initially unsympathetic to Ashya’s parents, but the spectacle of a very sick child being separated from his parents decisively turned public sentiment. Ultimately, the case was dropped and Ashya received proton beam therapy.

In *Roberts* and *King*, the Internet and social media were minor factors in each case. However, in subsequent cases, the potential of social media to publicise cases and mobilise support was more decisively realised. The first of these cases was *Gard.*^70^ This case concerned eight-month-old Charlie Gard, who suffered a rare and fatal genetic disease, for which his parents sought an experimental treatment. After suffering a prolonged spell of intractable epileptic seizures that severely damaged his brain, the treating hospital sought permission from the courts to remove life support. His parents opposed this and sought to transfer Charlie to the care of a US specialist who would provide their preferred treatment. Once they publicised the case on social media, it quickly became a media sensation, resulting in the accumulation of considerable funds from online donations and the sale of campaign merchandise.^71^ There were also interventions from international figures such as the Pope and the President of the USA, as well as many private individuals who gathered to protest outside the courts and hospital. Besides expressions of solidarity, this febrile atmosphere also had toxic effects, particularly on staff at the treating hospital who complained of abuse and death threats.^72^ Charlie’s parents eventually lost their case, but within months two further cases—*Haastrup*^73^ and *Evans*^74^—proceeded through the courts.

*Haastrup* concerned an 11-month-old boy. Isaiah Haastrup had been born by emergency caesarean section due to a uterine rupture, he required aggressive resuscitation at birth, and sustained severe brain damage. The circumstances of Isaiah’s birth led to a complete breakdown of trust between his parents and the hospital, who advised that further treatment was futile. A social media and crowdfunding campaign were launched, but received less attention and raised vastly fewer funds than the crowdfunding campaign initiated in *Gard*. This was in sharp contrast to the case that took place almost in parallel, *Evans*. This concerned 2-year-old Alfie Evans, a boy with an undiagnosed syndrome causing progressive developmental delay, epilepsy, and severe brain damage. The hospital sought to withdraw ventilation but was vigorously opposed by Alfie’s parents, who wanted Alfie transferred to an Italian hospital for further medical investigations and the continuation of life support. Alfie’s parents adopted a similar approach to that in *Gard*, aggressively using social media to engage supporters, raising funds via crowdfunding platforms and the sale of campaign merchandise. The case garnered global attention including that of influential figures, including the Pope and the Polish President. Protests took place outside the hospital and the court by members of the public mobilised through social media, who referred to themselves as ‘Alfie’s Army’. Hospital staff suffered online abuse, and protesters attempted to storm the hospital building.^75^ In both *Haastrup* and *Evans*, the hospital trusts were granted permission to withdraw treatment.

Most recently, *Raqeeb*^76^ concerned a 5-year-old girl, Tafida Raqeeb, who had suffered a catastrophic brain injury due to the rupture of a previously undiagnosed malformation of the vessels in her brain. The injury left her in a minimally conscious state and her doctors sought to withdraw treatment. As in *Evans*, Tafida’s parents sought to have her transferred to an Italian hospital for further investigation and establishment on long-term ventilation. The family used social media to publicise her case and raised funds via crowdfunding, and the case also saw the intervention of international figures, including the Bangladesh High Commissioner to the UK. At the time of writing, they remained short of their funding goal, despite winning their case in the High Court, although other sources of private funding had been reported in the press.^77^

When critically analysed, the *Gard* and *Evans* social media campaigns reveal a close similarity in the methods that were adopted. We speculate that *Evans*, coming one year later, looked to replicate the success of the *Gard* campaign by using similar strategies. Nevertheless, each of these cases has revealed the potential of social media to be a significant feature in decision-making for critically ill children in the future. While this potential was noted by Francis J in *Gard*,^78^ it is not to say that social media will directly influence a judgment (as Francis J was careful to stress).^79^ Rather, the sophisticated adoption of public relations teams and the financial resources available in *Gard* and *Evans* have had a considerable impact on the framing of subsequent cases both in policy, in clinical, and in legal practice. In policy, these two cases have become touchstones for calls for greater parental rights in English law.^80^ In clinical practice, the desire to avoid the type of hostile public involvement seen in *Gard* and *Evans* is likely to give clinicians pause in future cases, making them less willing to challenge parental demands. In legal practice, changes in clinical behaviours will ultimately change the types of cases that are seen by the courts, but more immediately the impact of *Gard* and *Evans* has already reduced the degree openness shown by the courts when they do appear. Soon after *Evans*, another case concerning the withdrawal of treatment from a critically ill child saw an NHS trust seek anonymity from the courts, arguing that the trust and the staff treating the child would be subject to the same vitriol as evident in *Gard* and *Evans*. The court authorised the withdrawal of life-sustaining treatment and ordered the name of the trust to be suppressed. In a private hearing, Moor J was reported as stating:

… these type of cases” raised “intense emotions” among people unconnected with day-to-day care. He said Great Ormond Street staff had been subjected to “very significant harassment” as a result of the Charlie Gard Case.^81^

Meanwhile, in the recent case *Re M (Declaration of Death of Child)*,^82^ concerning a brain dead baby, McFarlane P signalled flexibility in naming expert witnesses, acknowledging the potential for harassment by citing *Gard* and *Evans.*^83^ Further, the judge discussed reporting restrictions orders, noting that social media continue to be deployed in cases concerning disputes about treatment for children and the negative impact it can have on healthcare practitioners. He argued that openness and transparency in such difficult and often controversial cases needed to be balanced with the protection of those caring for the sick child.^84^ These developments took place against growing unease at the lack of openness of the Family Division. Its former President, Sir James Munby, recently called for a repeal of the current reporting restrictions in certain types of cases. He commented that the combination of the withdrawal of legal aid from many private law matters (due to the Legal Aid, Sentencing and Punishment of Offenders Act and section 12 of the Family Justice Act) had effectively made the Family Courts a lawyer and reporter free zone. In combination with the lack of published first instance judgments available, he argued that a concerning lack of transparency was ensuing.^85^ Thus, judicial responses to this aspect of globalisation, while understandable, are arguably worsening a crisis in access to justice and transparency of the Family Courts, a crisis which has potential to corrode confidence in the legal process at large.

#### 1. Social Media, emotion, and global activism

Social media, while undoubtedly a valuable panacea to the social isolation of caring for a child in hospital, also provides a global virtual space for likeminded parents, and other actors, to challenge medical and legal decisions in the public arena.^86^ Yet what is clear is that the strategies used in *Gard* and *Evans* did not just harness the Internet as a means to transmit ideas and information—the so-called first-generation of Internet technologies. They also used the potential of the Internet’s second-generation social media technologies to interact with the public on a raw emotional level. Indeed, as an aspect of globalisation, it was the ability of social media to rapidly cultivate collective emotion and focus it in hostile international public campaigns against hospitals and staff^87^ that is its most glaring impact. This aspect of emotional contagion is a radically different challenge to that which is represented by the unmediated flow of information alone. Social media allows users to surmount the hurdles of time and space that otherwise reduce emotional involvement with distant events and people. It fits within a pattern of globalisation by allowing third parties with little personal connection to events save personal sentiment, to lend personal support, in real-time, to specific parties in a dispute that is taking place in a foreign jurisdiction thousands of miles away. To a great extent, the ability of social media to cultivate and focus raw emotion has been one of conscious design.^88^ As one Facebook insider has written:

Facebook’s advertising business model depends on engagement, which can best be triggered through appeals to our most basic emotions. … emotions such as fear and anger produce a more uniform reaction and are more viral in a mass audience. When users are riled up, they consume and share more content. … The [Facebook] algorithms choose posts calculated to press emotional buttons because scaring users or pissing them off increases time on site.^89^

While social media can and is used to share facts and arguments, it more often fits the pattern of a ‘blind-conviction machine’^90^ due to its ability to affirm, harness, and amplify emotional responses to an issue. Studies of Twitter indicate that as few as 40% of social media consumers read beyond the headline of news items they share,^91^ and that Tweets featuring moral sentiment are more widely shared than those featuring intellectual engagement.^92^ A paradigm example of the way social media encourage emotional, rather than reflective, engagement with content is Facebook’s adoption of emoticons. On the massive scale of a global audience, these convey raw social approval or disapproval in a way that positively shuts down opportunities for discussion and reflection. As philosopher Michael Lynch opines, ‘if you use the angry emoticon … it is extremely unlikely that you will then comment by saying that the piece in question really made you think’.^93^

Children, of course, are a key focus of emotional sentiment. The effect of this globalised virtual space, which by design seeks and amplifies emotional responses, is that localised disputes about particular children can become magnets for popular sentiment. From there, sections of the global public are drawn into virtual spaces, such as the Facebook groups seen in *Gard* and *Evans.*^94^ Administrators of the groups only permit entry after specific questions are answered.^95^ This reinforces the ‘echo chamber’ effect to attract like-minded followers, which, even without the raw emotions that underlie their engagement, would leave little room for dissent. At the same time, these echo chambers inform wider media coverage, which disproportionately focuses on Twitter trends. The social media strategies employed in these cases included the use of nuanced ‘hashtags’^96^ that gained traction in the mainstream media after being frequently shared by supporters. The administrators of the social media groups uploaded footage, news updates, and daily photographs, creating a system of almost entirely ‘self-reported’ news that allowed direct control of the narrative. This further rallied support on a global scale,^97^ including influential public figures^98^ and third-party activists, making each child a potent symbol for globalised ideological confrontations.^99^

Third parties in different geographical locations used the anonymity of virtual spaces to discuss, comment, and advocate for causes and issues that were otherwise unlikely to have had wide public support in the ‘real world’.^100^*Gard* and *Evans*, in particular, served as springboards for social, political, and religious activism via the Internet,^101^ where social media was utilised ‘to misunderstand and misinterpret evidence, science, and law to malign public institutions’.^102^ For example, some commentators noted connections between activism in *Gard* and *Evans* and populist campaigns that are critical of communalised public healthcare systems like the NHS, including US political opponents of the Affordable Care Act (so-called ‘Obamacare’).^103^ Such trends highlight the opportunistic interlinking of political and social movements around science and healthcare issues under the current wave globalisation.^104^ The ability of social media to globalise emotional engagement has thus shown an ability to affect the decision-making environment in tangible ways: from fomenting unrest on the streets to influencing practice and policy. But perhaps just as importantly, this emotional outpouring can be turned into funds that can be used by parents to participate in a global healthcare market.

#### 2. Crowdfunding: turning emotion into money

A final aspect of the impact of the social media is access to crowdfunding. Crowdfunding is used as an online platform for raising money for targeted campaigns via specific websites dedicated to this purpose. The success of crowdfunding campaigns may be based on ‘capitalising on emotionally appealing stories and evoking empathy’^105^ making crowdfunded campaigns—especially those focused on children—uniquely suited to the workings of social media. Crowdfunding is increasingly being utilised as a means of raising funds for medical treatment.^106^ Overtly this was the role of crowdfunding in the cases discussed, despite access to funding only being at issue in *King*. Nevertheless, the families in *Roberts*, *Gard*, *Haastrup*, *Evans*, and *Raqeeb* all set up crowdfunding accounts. While substantial funds were raised in some campaigns, others were less successful. Potentially in earlier cases, this was because social media use was below a threshold level, and the algorithms that track emotional states less developed, to allow the sort of emotional connections seen in *Gard* and *Evans*.^107^ It remains a subject of speculation as to why *Haastrup* and *Raqeeb* saw less success. One explanation is that the campaign in *Raqeeb* was apparently conducted in a more sober manner than that in *Gard* or *Evans*, making it far less impactful if, as we assert, crowdfunding trades on emotional impact. Nevertheless, it may simply be that the case did not gain the initial traction to produce that impact. This suggests a second, more uncomfortable explanation that also accounts for *Haastrup*, that the participants in these cases did not elicit the empathy on which emotional impact relies. A growing body of experimental science suggests that we feel greater empathy with those whose affiliations we identify with. It is striking that both Isaiah Haastrup and Tafida Raqeeb were from ethnic minority communities and in Tafida’s case, a religious minority, so may have not have evoked the same visceral emotional connection among the segment of the population who felt so connected to *Gard* and *Evans*.^108^

There are advantages of crowdfunding, and raising awareness of rare medical conditions was often cited in the *Gard* case.^109^ Nonetheless, crowdfunding in healthcare raises ethical, social, and legal implications.^110^ Concerns identified with crowdfunding using critically ill children include issues discussed in relation to children’s privacy^111^ and disclosure of medical information by parents.^112^ There are more general concerns about fraudulent websites being created to raise funds; the costs, fees, and waivers demanded for the usage of crowdfunding websites; and the crowdfunding websites’ power and control in choosing which causes and campaigns to support and/or reject, driven by market values and attitudes of the day.^113^ Despite these issues, successful crowdfunding allows parents to participate in a global healthcare market that may otherwise be beyond their means. This highlights the second way that globalisation raises challenges to decision-making for critically ill children: access to innovative or novel treatments.

### B. Parents Seeking Innovative or Novel Medical Treatments

While social media present significant challenges, these have become particularly pertinent when combined with parental requests for alternative medical treatments. Proposals for alternative treatments with no proven medical efficacy have been considered sporadically in cases concerning children in the past,^114^ although consideration of treatments where there was a level of medical disagreement about efficacy have been rare.^115^ However, an increasingly globalised approach to healthcare has meant a growing trend towards novel treatments or therapies where there is some level of support from medical professionals internationally. Many of these therapies might be classed broadly as novel or innovative, yet such labels may be unhelpful as they are often used rhetorically.^116^ A more expansive typology suggests three cases where we might consider medical treatments novel:

1. Benefits and risks are hypothetical since the treatment is entirely experimental.
2. Treatments are of unproven effectiveness and awaiting/still undergoing research trial(s).
3. The balance of risks, benefits, and efficacy means treatment is not commonly used for the purpose for which they are sought.

#### 1. Entirely experimental treatments

Although the courts had first considered the issue of a child receiving an entirely experimental treatment in *Simms*,^117^ it was not until *Gard* that the issue was considered with the cross-border elements that fit the rubric of globalisation. *Gard* shared many of the features apparent in *Simms*—both considered progressive and fatal diseases, where experimental treatment with a related disease had signs of efficacy in animal research.^118^ Additionally, in *Gard*, unpublished human data from a safety trial in the related condition seemed promising.^119^ Yet crucial differences in this related condition—which, unlike Charlie’s condition, did not affect the brain—underlined the experimental nature of the proposed therapy. Once the severity of Charlie’s brain damage was accepted by all parties, the case was abandoned.^120^ Published research on Charlie’s mitochondrial disorder remains focused on describing the mutation and its variations.^121^ Its rarity means that any treatments can be presumed to remain highly experimental.

#### 2. Treatments of unproven effectiveness

In *Roberts*, Neon’s mother wanted her son to receive several alternative treatments that range across our typology. These included diet and lifestyle therapies, entirely experimental treatment,^122^ and treatment not commonly used for the purpose proposed.^123^ Finally, treatments of unproven effectiveness were also requested, including proton beam therapy, which we discuss below.^124^ The overall impression was a scramble for any imaginable alternative treatment, with little consideration of the likelihood of benefit.

The parents in both *Roberts* and *King* requested access to proton beam therapy, for which there was no evidence of additional benefit at that time. In *King*, proton beam therapy was eventually given, apparently successfully. By 2018, Ashya had returned to school, with scans showing no signs of a recurrence of cancer.^125^ A 2016 study on the effectiveness of using proton beam to treat Ashya’s type of cancer^126^ suggested proton beam therapy has fewer side effects than conventional therapy^127^ and was widely seized upon as a vindication of Ashya’s parent’s position.^128^ Proton beam centres were rapidly established in the UK,^129^ and current guidelines for clinical commissioners judge the evidence for routine commissioning to be sufficient.^130^ Nevertheless, evidence of benefit remains relatively weak^131^ and the explosion of centres globally has been criticised by proponents of evidence-based medicine.^132^

#### 3. Treatments not commonly used for the proposed purpose

In *R v Cambridge*,^133^ the father of a 10-year-old sought further treatment for leukaemia after a third recurrence. An expert in the field agreed that further treatment might be appropriate, but no clinician who was willing to offer treatment on the NHS was found. Private treatment was available, both in the UK and internationally in the USA, but prohibitively expensive. The health authority categorised the treatment, which had a 10–20% chance of success, as ‘experimental rather than standard therapy’,^134^ and would not fund it. The court of appeal upheld this decision.

In the cases of *Haastrup*, *Evans*, and *Raqeeb*, surgery, including the formation of a gastrostomy to allow feeding and a tracheostomy through which they might be ventilated, was sought to allow the child to be cared for at home.^135^ In each case, the UK, and sometimes international, medical teams argued that the surgery was not appropriate given the severity of each child’s condition. The controversy over the use of ‘home ventilation’ in medically futile cases centres on the putative damage to the child and family’s interests in providing the treatment, which has, in more favourable circumstances, been described as a ‘complex tension between … distresses and enrichments’.^136^ Home ventilation through the use of small, portable ventilators, while technically feasible, remains uncommon, partly due to prohibitive costs.^137^ In the UK, it is still an exceptional therapy^138^ rather than a routine measure and remains controversial when it will not be a therapeutic bridge towards a cure. Nevertheless, healthcare professionals who would be willing to sanction such treatment were located internationally in each case. Whether and when home ventilation should be offered to families remains a topic of intense debate.

#### 4. Request for novel treatments by parents of critically ill children

The case of treatments being requested outside circumstances where doctors in a particular jurisdiction consider that they would not be effective raises questions about the scope of considerations that should inform medical decision-making. While controversies about the value-laden nature of ‘medical futility’ are well explored, cases such as the (non) provision of home ventilation seem to turn as much upon considerations of the fair distribution of limited healthcare resources. In a public healthcare system, where risks are communalised, such considerations are generally not a focal point when determining end-of-life treatment decisions in individual cases. Nevertheless, it has been argued that where resources to access innovative treatments are not at issue, and such treatment(s) are affordable, they should not be denied, as they do not prevent other patients accessing conventional, publicly funded treatment.^139^ This has especially been a point of contention where parents have raised substantial funds through crowdfunding, and it was nonetheless held that treatment was not in the ‘best interests’ of the child.^140^ Some commentators have argued that this analysis is too simplistic and does not properly account for the costs of maintaining a healthcare infrastructure.^141^ In the UK context, it has been argued that the de facto inequalities in spending that will arise from such an approach may make it publicly divisive.^142^ These issues of just resource allocation are also raised by increasing access to treatments that have no established efficacy. It has long been noted that the headlong rush to expensive, cutting edge therapies by healthcare providers often precedes any convincing evidence.^143^ By the time better evidence is available, investments have been made that commit healthcare providers to these technologies even if the evidence does not bear out initial optimism.^144^ Such a dynamic seems ever more likely when the spending decisions of healthcare providers are guided by public opinion rather than scientific evidence.

Ongoing developments in medical research, particularly in the areas of stem cell and gene therapy, continue to raise hope of a cure to patients suffering progressive and/or incurable medical conditions.^145^ The continuum of experimental, unproven, and non-indicated treatments highlights the fact that there is no clear line between routine and novel treatments. Standards of proof may be relative rather than absolute.^146^ Given the willingness of parents to seek novel treatments even when unproven and/or in the early clinical trial stage, ethical concerns have been raised about how and when novel treatments should be offered to critically ill children.^147^ Some have argued that treatments with very low chances of success should be discussed as ‘fantasy treatments’ to better communicate these small chances.^148^ Yet it seems unlikely that challenges are merely or substantially linguistic. Gross differences in interpretation can occur even where evidence appears overwhelming.^149^ In such cases, hope, however unrealistic, may be the natural reaction of ‘loving parents who are simply battling to stay upright in the darkening storm that has overwhelmed their family’.^150^ The huge spectrum of medical opinion accessible in a global market means that increasingly families whose hopes may be considered unrealistic will find some doctors who are willing to provide treatment. In such a scenario it is clear that there is a role for the courts in determining both the quality of treatment as well as the admissibility of the values that underlie both the request and the reasons for accepting the patient.

How this balance between values and evidence is to be achieved is not always clear at the outset of a case. Arguably the tension might in part be removed by a clear statement of what would be required before such a request would be accommodated, such as Bodey J did in *Roberts* when he stated that an acceptable treatment must be adequate on the grounds of evidence, expertise, and infrastructure.^151^ Yet the question of which values the court should find acceptable is more thorny. The courts have yet to alight on a settled approach, with some cases where novel treatment is sought giving clearer weight to values—for example, *B v D*^152^ and *Raqeeb*—and others—such as *Gard* and *Evans—*where evidence predominates.

At the nexus of networks of information, emotional connection, funding, and access to novel treatments lies the third, and least examined challenge of health globalisation in children’s healthcare: child medical tourism.

### C. Child Medical Tourism

Accessing global markets in medical treatment entails travel to different jurisdictions. Where children are involved, this phenomenon has been referred to as ‘child medical tourism’.^153^ The expansion of child medical tourism as an element in recent cases appears to have been catalysed by the supporting elements of well-developed Internet and social media platforms, which allow parents to widely publicise their cases, build emotional connections through social media, seek alternative treatments and providers, and to raise funds. Although travel was not at issue in *Roberts*, Neon’s mother’s open letter to the British Prime Minister^154^ indicated a wish to take Neon abroad for treatment. In *JM*,^155^ the family of a 10-year-old boy with an aggressive cancer of the jaw returned to their native Poland after refusing to consent to conventional therapy. Despite doctors advising that non-treatment would inevitably be fatal, his parents sought treatment of the cancer with alternative means.^156^ The family could not be traced in Poland, effectively curtailing further intervention despite the English courts formally retaining jurisdiction in the case. In *King*, despite advice that proton beam therapy would give no clear advantage over conventional treatment for Ashya’s cancer, his parents nevertheless located a hospital in Prague willing to give Ashya the therapy. Following the family’s flight, the parents put forward a ‘coherent and reasonable’^157^ treatment plan. The UK government intervened, making funds available for Ashya to receive the treatment in Prague.

While Ashya’s medical needs meant that travel was felt to place him at a risk of significant harm, recent cases have involved children so unwell that only a medical transfer would be possible. In *Gard*, once the hospital formed the opinion that pursuing experimental (or any) treatment was no longer in Charlie’s best interests, his parents sought to travel to settings outside the UK willing to provide the experimental therapy. They received offers from both a New York hospital and the Vatican hospital, and Charlie was granted permanent US residence to expedite travel to the USA. In *Haastrup*, while there were no overt plans for international travel, his parents’ legal team obtained an expert opinion that Isaiah was fit to travel to Germany. *Evans* again saw the development of detailed plans for treatment outside the UK. While Alfie’s doctors sought permission to withdraw treatment, Alfie’s parents wanted him transferred to the Bambino Gesù Hospital in Rome, where he had been accepted for further medical investigations and the continuation of life support. Following the failure of their case in successive courts, including earlier application to the European Court of Human Rights, Alfie’s parents sought a hearing from that court on the basis that preventing Alfie’s transfer to Italy was a violation of his Article 5 convention rights to liberty and security. In the interim, Alfie was granted Italian citizenship by the Italian ministry of foreign affairs, in the hope, this would facilitate his transfer.^158^ In the event, the European Court ruled the application inadmissible.

*Raqeeb* saw a similar emphasis on rights to international transfer. Having secured agreement to treat Tafida from healthcare professionals at Gaslini Hospital in Genoa, Italy, her parents took the unusual step of seeking judicial review, allowed under the NHS Patient’s Charter, of the hospital’s decision to refuse to allow her to be transferred, while Tafida’s best interests were in dispute. As a result of the review, the judge ruled that the hospital had not considered Tafida’s Article 56 rights under the Treaty for the Functioning of the European Union (EU), to access services within the EU. Failure to consider their obligations had effectively breached those rights. Nevertheless, if the hospital had correctly considered these obligations, it would have been apparent that sufficient public policy grounds existed to prevent Tafida’s transfer to Genoa, while her best interests were contested. In other words, the hospital had erred, but ‘would … have arrived at precisely the point it has now reached’.^159^ had they acted correctly. In the event, the judge found that withdrawal of treatment was not in Tafida’s best interests, and she was transferred to Genoa. Her parents are reported to be seeking Italian citizenship.^160^

In all of these cases, the parents of the critically ill children sought treatment outside the UK, and/or second opinions from overseas specialists to obtain putatively better treatment options that were contrary to the advice of their healthcare teams. The recent upsurge in cases involving cross-border issues appears significant. Certainly, some early cases involved international travel as a potential issue: in *R v Cambridg*e,^161^ the patient’s father made contact with US clinicians who were supportive of treatment. Yet the lack of developed Internet meant limitations on the types of treatments that could be researched, while absent social media meant neither were there means to elicit wider emotional connection and support nor that the costs involved could be overcome by crowdfunding. It seems only now that the key elements are in place that child medical tourism can emerge as a significant issue. Despite its growing importance, there is very little research that explores child medical tourism, defined by one source as ‘the bi-directional movement of children (less than 18 years of age) to and from a country to seek advice, diagnosis and treatments’,^162^ although medical tourism involving adults is better explored. The literature available has identified several different forms of child medical tourism. These include: parents from low resource countries wishing to access overseas healthcare for their children, funded either privately or by humanitarian organisations; parents from wealthy countries also travelling overseas. This might be to obtain healthcare that is unavailable in the UK, or is unavailable under the NHS but accessible through reciprocal healthcare arrangements, or that they believe to be of a higher quality.^163^ Further, parents may also opt to travel overseas for cultural reasons, in some instances travelling to countries with fewer healthcare resources.^164^

The case most familiar to English medical law relates to parents in high-income countries seeking treatments from other high-income countries that are unavailable in their home country. Such scenarios appear only rarely in accounts of medical-legal disputes,^165^ more often being encountered as uncontested decisions by parents reported in the news media.^166^ Indeed, as we have noted, some may view access to overseas treatment, using private funds, as a quintessential example of parental rights to exercise health choices on behalf of their child. They may argue that the choice is all the more morally laudable because it removes pressure from the NHS infrastructure.^167^ Similarly, judges may see it as beyond the ambit of their powers to make judgments as to the fitness of practice of doctors in a faraway country. Certainly, the Family Court has taken such an attitude to differences between national approaches to children’s social welfare.^168^ As the former President of the Family Division, Sir James Munby recently wrote in relation to a failed case that sought to remove children from their parents who were being deported to a country from which they had sought asylum, the only reason to intervene in such a case was ‘because, at root, we are not prepared to trust the public authorities of the other state’, concluding ‘… what business is it of ours?’.^169^

Such comments point to a wide degree of parental discretion in social choices. Nevertheless, there are putative differences between the evidential bases of medical and social welfare.^170^ The duties of public authorities in other jurisdictions may be quite different from the standards we might expect from doctors, whose professional judgments about offering treatment should arguably be led by a rational consideration of evidence, risks, and effectiveness. While evidence transcends international borders, it is not clear that we can always establish clear distinctions between environmental and medical factors. Medical cases are not decided on facts alone and have the potential to raise their own serious ethical issues depending on cultural and legal differences between the destination and the originating country, both in the way children are viewed and the way healthcare is delivered. For example, the extent to which jurisdictions see children as appendages of their parents rather than independent rights holders, or the degree to which requests for treatment are limited by the ability to pay may engender different expectations of the parental role in the decision-making process, the balance of harms and benefits to the child, and degree of support available for long-term outcomes. Certainly, arguments citing national differences in medical perspectives were advanced in *Gard*, where one British doctor noted the ‘cultural difference’ in philosophy between treatment in the USA and in the UK. She stated that she ‘tried to have the child at the centre of her actions and thoughts whereas in the United States, provided there is funding, they will try anything’.^171^ Moreover, globalised medical care cuts both ways. Since medical tourists have no impediment to their right of return to their originating country, the impact of a treatment decision is not simply confined to distant jurisdictions but may have direct repercussions on the originating country. For example, studies of complex interventions have documented the potential for deleterious impacts on parental well-being.^172^ We have already noted the ‘enormous caring commitment’ shouldered by parents and siblings of home ventilated children.^173^ The pressures of these commitments may create a need for both inpatient and respite support. Failure to provide this may result in increased mental ill-health and family breakdown.^174^ Such knock-on effects have not been addressed by the courts, although they may conceivably fall within the ‘best interests’ determination given the focus on the quality and long-term effects of treatment.

While child medical tourism is almost entirely invisible and largely unstudied, adult medical tourism is a multi-billion pound sterling growth industry.^175^ It has been noted that there are globally approximately five million people that travel annually to another country paying for medical treatment as an out of pocket expense.^176^ While separate, we can draw parallels between the adult and child phenomenon. The growth of medical tourism worldwide has been primarily driven by fierce Internet and social media marketing and low-cost medical treatment that is supported by inexpensive travel.^177^ Despite the heterogeneity of medical tourists, concern has been expressed in many sectors about negative impacts on patient care for returning medical tourists. This arises through a lack of continuity and breakdown of patient–practitioner trust, unclear boundaries of medical responsibility and difficulties in enforcing legal liabilities across international boundaries, privacy and confidentiality of medical information, timely sharing of medical information between countries, and differing standards of care.^178^ While some progress has been made in international accreditation between particular nations and in discrete clinical specialties, such as the accreditation of heart surgery in India for US patients,^179^ such accreditation is piecemeal.^180^ Indeed, accreditation agreements, while increasing patient choice, still place distinct limits on patient liberties, especially if they are seeking novel treatments that are not widely recognised or approved. Significant numbers of patients travel abroad to circumvent prohibitions on treatments in their originating country,^181^ while others travel abroad for unproven treatments for diseases for which there is no conventional cure, such as multiple sclerosis.^182^ Since similar motivations lie behind the majority of instances of child medical tourism, the challenges of medical tourism thus appear magnified in the case of children.

## V. IMPLICATIONS OF HEALTH GLOBALISATION ON CHILDREN’S MEDICAL TREATMENT

We have discussed a series of issues that we argue are the signs of increasing globalisation in children’s medical treatment. These issues present growing challenges to the current clinical practice, and ultimately, to the operation of the law in this area. These are interrelated challenges and we argue that it would be a mistake to view them in isolation. The impact, on numerous levels, of the Internet and social media on the decision-making process, by providing ready and unfiltered information, creating emotional connections with large numbers of third parties across the globe, and ultimately through access to crowdfunding, has been instrumental in providing access to innovative or novel treatments. In most of the cases we have discussed, providers of these treatments were located in a second country. This internationalisation of cases raises the issue of child medical tourism as a distinct issue emerging from the wider phenomenon of medical tourism at large. Because these are interconnected challenges, none can be satisfactorily addressed without consideration of the other. For example, action on medical tourism requires meeting the challenges of unmediated access to unfiltered information about innovation, as well as the design aspects of social media that encourage an emotional response from third parties to highly complex situations. Similarly, regulation of innovation requires the forging of common medical standards that will ultimately play a role in protecting medical tourists, including children. Conversely, any of these responses that are taken in isolation from any other will be rendered less effective. The challenges of globalisation require a comprehensive and multi-layered response.

Detailing such a response is a task much greater than we are able to achieve in the space of this article alone. Nevertheless, as our discussion so far has highlighted, both the impact of the Internet and access to innovative treatment have drawn the attention of commentators,^183^ but child medical tourism has, as yet, been relatively neglected. In order to begin to rectify this research deficit, we will set aside the comparatively well-trodden topics of the Internet and innovative treatment to highlight a number of potential remedies and directions for future research to meet the specific challenges of child medical tourism. These address challenges to a child’s protective rights under the UNCRC, the difficulties in enforcing private contracts across national boundaries causing a similar erosion of the rights of parents as consumers of healthcare services, and putative economic impacts for national healthcare systems.

###  

#### 1. Challenges to the protection of children’s rights

Arguably, the negative impacts of globalisation on children at large may be mitigated by the economic benefits of market liberalisation of which a growing health tourism sector is a part. Economic benefits are vulnerable to the cycles of economic recession. Yet, so long as economic growth is present, mainstream economic theory suggests that growth is likely to improve the basic welfare of children in low- and middle-income countries, thus furthering their Article 27 UNCRC rights.^184^ Growth raises the floor of living standards by both enabling an enriched state to provide better services, and by raising family income. Unfortunately, while sometimes visible, these benefits are not seen uniformly—trade inequalities^185^ and corruption^186^ in some poorer countries have been argued to exceed gains to welfare, while the hijacking of globalisation by special corporate and ideological interests have been argued to have done much to mute its potential to benefit.^187^ To the extent that economic benefits can be realised, they underline the need for a nuanced response to concerns about the negative impacts on children in wealthier countries as inbound health tourists. Nevertheless, the duty of ratifying states to fulfil their Article 27 obligations must coexist with their other obligations under the UNCRC, including Article 3. Despite the almost universal ratification of the UNCRC, it may be too much to expect the rather broad terms of Article 3 to be in themselves protective of child medical tourists because the incompletely theorised nature of these rights allows significant leeway for interpretation of the convention by ratifying states.^188^ Moreover, it is clear that parents themselves also enjoy strong rights under the convention. Thus, children appear at risk in two ways. First, while the UNCRC recognises that first and foremost it is parents who are responsible for the care of their children,^189^ parents may lack adequate information to choose healthcare providers responsibly. This appears likely if the standards and patterns of information provision in child medical tourism mirror those in the adult case. These problems may be mitigated if choices are made in consultation with a professional in the UK who will be able to give an independent and expert assessment of whether what is being offered is trustworthy and of a sufficient standard. Yet the influence of this assessment may be negligible in those cases where parents are seeking overseas treatment because of a breakdown in the partnership with healthcare professionals in their home country. To some extent, the role of the courts in the cases examined in independently scrutinising the quality of treatment being offered therefore seems justified. Of course, further research is needed to determine if the process could be streamlined, and if so, whether such streamlining could successfully take place using current, largely informal, networks of second medical opinions.

A second, more serious, concern is raised by what happens to children after their arrival overseas. Given the wide cultural variations in the perceived status of children, they face the prospect of travelling to jurisdictions where their rights are de facto synonymous with the choices of their parents, and these choices are part of a transaction that emphasises the commercial, rather than fiduciary, aspects of providing medical treatment. Thus, children risk being reified by healthcare providers who entirely defer to the choices of parents. This stance becomes especially problematic when parents are purchasing treatment that is unnecessary, suboptimal, or harmful to the child, for example, where parents seek female genital cutting or ‘conversion therapy’ aimed at changing a child’s sexual orientation. Medical tourism undertaken expressly to avoid legal prohibitions of a medical procedure in a country of origin by travelling to a destination where the procedure is legal has frequently been reported. Cohen has coined the term ‘circumvention medical tourism’ to describe the phenomenon.^190^ As he explains, the prosecution of parents for circumvention tourism is possible in the country of origin under international law if it comes to light. Nevertheless, while it is likely that examples like female genital cutting represent undisputed, black and white cases of parental criminality, there is considerable legal greyness in many medical cases, for example, the actions of the parents such as those in *JM*,^191^ where an (arguably) suboptimal treatment^192^ may be being sought. It is unclear where the line drawn by the harm threshold in section 31(2) of the Children Act would fall in such cases. While rejecting a proposal to adopt a threshold of significant harm in a civil case, McFarlane LJ in *Gard* found that the continuation of intensive care to allow treatment of doubtful viability itself amounted to harm.^193^ The facts surrounding *King*, on the other hand, indicate that criminalisation of desperate parents, however misguided, is likely to arouse strong public feelings, and therefore seems neither assured in all cases nor, if it would entail the separation of otherwise responsible parents from their child, necessarily desirable. This issue raises complex questions about the most effective uses of criminal and civil law in this area, as well as a consideration of how the UNCRC can efficiently provide a counterweight to the reification of children in decisions about medical treatments. These require further research.

#### 2. Parental rights and difficulties in enforcing consumer rights to healthcare

While the risks to the interests of children in child medical tourism mean that concern may be aroused on children’s rights grounds, there appear equally strong reasons for concern among those who are more ambivalent about the status of children’s rights but favour strong parental rights to be health consumers.^194^ The commercial aspects of medical tourism per se may raise concerns about the inequitability of a system that excludes parents who lack the resources from accessing the overseas treatments they prefer. Even where this is not a concern, there are uncertain pathways to legal redress should treatment fail at any stage due to negligent performance. This should be a more unifying concern to those who view child medical tourism favourably. Although the recent departure of the UK from the EU makes it uncertain if current jurisdictional rules under the Brussels and Lugano Conventions will continue to apply, the situation is already uncertain if negligence happens outside the EU. Here, if a claim is pursued in the UK, the defendant may choose to contest the jurisdiction of the court^195^ and it may entail lengthy proceedings to establish jurisdiction before the substantive case is even engaged. If jurisdiction is theoretically agreed, it remains a potential obstacle that the court must be in the most suitable location to bring the action. In the case of children, *forum non-conveniens* has been held to be subordinate to the welfare principle, suggesting this particular hurdle may be slight.^196^ Nevertheless, the English courts will tend to follow foreign law, which may come with its disadvantages for the plaintiff parents.^197^ For example in *Naraji v Shelborne and Jari*,^198^ an action against an orthopaedic surgeon in the USA by a professional footballer failed in the English courts due to the shorter limitation period for personal injury claims in Indiana. Local laws in some medical tourism destinations have been noted to be unpredictable (eg United Arab Emirates)^199^ or deferent to doctors in key aspects of negligence claims (eg Malaysia and Singapore).^200^ A potential advantage of using the English courts is to avoid sometimes drastic caps on damages that operate in other jurisdictions potentially popular with medical tourists, such as Thailand.^201^ Yet, even when a judgment is reached in the patient’s favour, there may still be problems with enforcement. Destinations, where medical tourism is a major part of the local economy, may be reluctant to enforce damages.^202^ This is particularly likely where the amounts of damages awarded are unusually large for the provider country (which may be expected to be the case where damages compensate a child against lifelong harms), and where no reciprocal enforcement agreement is in place between the two jurisdictions^203^ (which may relatively frequently be the case, both internationally and within the EU).

Numerous local or multinational solutions have been proposed to improve the legal position. As we have noted above, systems, whereby destination institutions receive accreditation from entities in originating jurisdictions, have already been developed on a small scale. Similarly, some argue that expanding protections in originating countries, for example, by strengthening accreditation programmes by imposing legal requirements for them,^204^ or by reforming consumer protection law,^205^ is at least part of the solution. These reforms may have little impact where novel treatments are being sought. Arguably, more robust consumer protections lie in the development of international accords. Since existing trade accords tend to reduce rather than increase protections,^206^ new agreements would be needed, as well as bodies to police them. Podlaski^207^ proposes the creation of an international convention on patient rights, mirroring existing human rights agreements. Yet, while it addresses the unsatisfactory legal position head-on, such an agreement seems unlikely. Since medical tourism is a major source of revenue to several emerging economies, the ability to agree to such accords will depend largely on the ability of the originating jurisdiction to leverage their position as consumers. Greater leverage presupposes some way to influence the flow of health consumers, and this, in turn, requires governments to adopt a less laissez-faire approach to the global healthcare trade. Given the large flows of adult health tourists, the political will for effective change might indeed be found. However, the flow of child medical tourists may be too low, or too contentious (given the questions around children’s rights that we raise above), presenting obstacles for their inclusion in any potential agreement. Local laws need to be developed first in order to clarify national positions on child health tourism before serious work can begin at an international level.^208^ In the absence of this, it would seem prudent that child medical tourism is only undertaken when parents are fully cognisant of their precarious legal position should things go wrong, and only then with the active involvement of and support of recognised expert clinical teams in both the origin and destination countries who are well acquainted with the child. Such support does not necessarily mean a full agreement between both sets of healthcare practitioners but does imply that the approach taken is recognisably one that is medically reasonable. Again this is difficult in situations when there is a significant dispute between parents and healthcare practitioners, but not impossible. Of the examples we have considered, *Raqeeb* seems to have fulfilled this latter criterion, but further research is needed to develop a consistent approach.

#### 3. Economic impacts on national healthcare systems

A significant number of arguments for and against medical tourism are economic, and the economic impacts of child medical tourism invite similar debates. As we have noted, the impacts of medical tourism are nuanced, with the benefit of reduction in healthcare costs^209^ balanced against costs associated with complications necessitating further and/or ongoing treatment.^210^ An economic study by Hanefeld and others suggests that in the UK income and expenditure associated with inbound and outbound medical tourism result in neutral costs to the NHS.^211^

While any discussion of the economic impacts of child medical tourism is limited by the lack of available data, in the UK at least, these impacts are likely to both share similarities, and have strong distinctions, from adult medical tourism. Distinctly from its adult counterpart, child medical tourism is unlikely to have large economic upsides. It has yet to receive significant attention from institutional stakeholders, such as the NHS, to reduce costs and waiting times of routine treatment. While the actual scale of child medical tourism is unknown, its low profile suggests that it does not have an extensive international market like that of adult medicine. Negligible private traffic (in the UK at least) means that economic savings are similarly likely to be insignificant. However, it is likely that child medical tourism shares similar economic downsides to adult medical tourism, with children returning to their resident country facing similar hazards of infection and/or the need for corrective treatment. Indeed, potentially a child will continue to utilise NHS services for much longer periods than an adult. The growing numbers of patients with long-term conditions, both adults and children, who access innovative and unproven medical treatments overseas, present a risk of additional demands for services on their return, especially if the innovative treatments fail to be successful—or, if successful, result in large ongoing costs. It is unclear if the NHS would consider it had a duty to provide either remedial or ongoing treatment for patients who had been privately treated abroad. However, a Department of Health circular released following the PIP breast implant scandal indicated the NHS is likely to consider its role in ameliorating surgical mishaps in private patients to be strictly limited. In that case, the NHS was instructed to remove, but not replace, failing PIP breast implants in patients who had them implanted privately.^212^ It is unclear what this would mean for a child with significant ongoing health costs. In *Raqeeb*, the expert witness for Tafida’s family indicated that, should Tafida be established on a home ventilator, she would still require intermittent admission to intensive care to manage acute airway issues.^213^ The stated goal of Tafida’s treatment is that she be in a position to be cared for at home, and some, so far limited, progress has been announced in weaning her from permanent ventilation.^214^ Tafida’s parents have stated that their aim is to return to the UK.^215^ While any decisions about her treatment options would be made according to her best interests,^216^ should she return home with significant ongoing treatment needs there is potential for her entitlement to NHS care for ongoing treatment to be challenged.

The problem of repatriation of patients to resource-poor environments is a significant ethical and legal issue,^217^ but the potential flow of child medical tourists from private healthcare systems who will offer any treatment at a price, back to publicly funded systems for long-term care once the treatment is established, is as yet little studied or examined. Whether such problems grow in profile will to a large extent depend on the attitudes of the courts towards parents seeking to access globalised healthcare markets. The English courts to date have maintained a long-standing^218^ unwillingness to engage in an explicit discussion about the role, impact, and effect of resource limitations on treatment decisions.^219^ While judicial reluctance to engage with the significant short- and longer-term resources implications where disagreement occurs in the care and treatment of critically ill children is understandable, it is illogical to argue that it can be separated from the law where both the use of the courts, and the decisions they make, will have significant resource implications. Economic studies are a piece of the research jigsaw that is needed to inform the development of policy response to child medical tourism, and ultimately to the impact of globalisation on children’s medical treatment at large.

## VI. CONCLUSION

The process of globalisation presents enormous challenges not just to the law as it pertains to healthcare, but arguably to the nation-state itself. Few nowadays accept that there was ever a time when the nation state was truly unencumbered in its exercise of internal and external sovereignty,^220^ and, as Harrington^221^ points out, global interests need local infrastructure and the organisational apparatus that goes with a nation state. Nevertheless, such interdependence does not preclude challenges to systems of law that may ultimately weaken public confidence.

The increasing opacity of the Family Court in response to the globalisation of localised healthcare conflicts clearly has the potential to play to this narrative. Whether such a fatal undermining of the authority of the courts does take place is not, of course, solely in the hands of the Family Court. The issues attending globalisation are truly daunting. Yet while the Family Court does not face these challenges alone, it does have a part to play. Cases involving critically ill children are emotionally resonant and compelling—indeed, as we have argued, this is one reason why they have the potential to be globalised. The way that the Family Court responds to the effects of globalisation can undoubtedly play an important role in the unfolding narrative. The question is, what role? We agree that there is merit in engaging with each case primarily in terms of its individual complexity. However, perhaps the most damaging response would be to focus on each case as if it existed only within its peculiar bubble of individual factors, without engaging with the wider trends. While the wider policy context is a regular feature of reflection in discussions of the law, reflected here in recent debates about the transparency of the Family Courts, there is still significant scope for a more coordinated approach to be developed that confronts the multi-pronged sources of current challenges. We have signalled the way emerging trends and problems in children’s medical treatment appear connected to globalisation, and these problems need to be addressed in a coherent way that recognises their root causes, reaffirming a commitment to social, institutional, and economic justice, not just nationally, but an internationally. The solutions and related research agenda we have suggested in the final part of this article focus on the problems specifically arising from child medical tourism, but this should not detract from our central contention that child medical tourism lies at the nexus of global interconnectivity and the accessibility of novel and unproven treatments through a largely unregulated global marketplace. The law has a part to play here, both in an academic context by taking up the research agenda, and in practice by responding to the impacts of challenges in a coherent way where it can, and advocating for justice and reform where it cannot. There are signals that judges are growing attentive to some of these impacts. We must not underestimate the scale of these emerging challenges, nor of their challenge to effective governance and public confidence in the law.

## Footnotes

1 S Barclay, ‘Recognizing and Managing Conflict Between Patients, Parents and Health Professionals’ (2016) 26 Paediatr Child Health (Oxford) 314.

2 LK Fraser and others, ‘Rising National Prevalence of Life-limiting Conditions in Children in England’ (2012) 129 Pediatrics 923.

3 J Newman and E Kuhlmann, ‘Consumers Enter the Political Stage? The Modernization of Health Care in Britain and Germany’ (2016) 17 Journal of European Social Policy 99; K Veitch, ‘The Government of Health Care and the Politics of Patient Empowerment: New Labour and the NHS Reform Agenda in England’ (2010) 32 Law & Pol’y 313; T Borkman and C Munn-Giddings, ‘Self-help Groups Challenge Health Care Systems in the US and UK’ (2008) 10 Adv Med Sociol 127.

4 KJ Lindley, D Glaser and PJ Milla, ‘Consumerism in Healthcare Can Be Detrimental to Child Health: Lessons from Children with Functional Abdominal Pain’ (2005) 90 Arch Dis Child 335.

5 G Birchley and others, ‘Best Interests’ in Paediatric Intensive Care: An Empirical Ethics study’ (2018) 102 Arch Dis Child 930.

6 See, *Re C (Baby: Withdrawal of Medical Treatment)* [2015] 150 BMLR 161; *An NHS hospital Trust v HK* [2017] EWHC 1710 (Fam); *King’s College Hospital NHS Foundation Trust v Thomas (Withdrawal of Treatment)* [2018] EWHC 127 (Fam).

7 See *Portsmouth City Council v Naghmeh King & Ors* [2014] EWHC 2964 (Fam); *Great Ormond Street Hospital v Constance Yates & Ors* [2017] EWHC 972 (Fam).

8 See *King*, ibid; *Alder Hey Children’s NHS Foundation Trust v Thomas Evans & Ors* [2018] EWHC 308 (Fam); *Barts NHS Foundation Trust v Raqeeb* [2019] EWHC 2531.

9 See *Manchester University Hospital NHS Foundation Trust v M & Anor* [2019] EWHC 468 (Fam); *NHS Trust v BK and Others* [2016] EWHC 2860.

10
*Glass v United Kingdom* (2004) 39 ECHR 15.

11 JA Harrington, ‘Law, Globalisation and the NHS’ (2016) 31 Capital & Class 81.

12 R McNamee, *Zucked: Waking Up to the Facebook Catastrophe* (Penguin Press 2019) 69.

13 art 3(1).

14 CA89, s 1(1).

15 art 18(1).

16 CA89, s 3(1).

17 For the origin of this principle in common law, see Lord Scarman’s speech in *Gillick v West Norfolk and Wisbech AHA* [1986] AC 112, 184.

18 J Bridgeman, ‘The Provision of Healthcare to Young and Dependent Children: The Principles, Concepts, and Utility of the Children Act 1989’ (2017) 25 Med Law Rev 363.

19 This approach affords a series of ‘checks and balances’ where doctors may offer medical treatments but not proceed without parental consent, and parents may refuse consent but not demand alternative treatments. See: *Re J (a Minor)* [1991] Fam 33, [41] (Lord Donaldson MR).

20
*Glass* (n 10).

21 There is no published activity in this area before the criminal case of *R v Arthur* [1981] 12 BMLR 1.

22 See R Huxtable, *Law, Ethics and Compromise at the Limits of Life: To Treat or Not to Treat?* (Routledge 2013). See also M Brazier and E Cave, *Medicine and the Law* (6th ed, Manchester UP 2016) 442.

23 J Bridgeman, ‘Gard and Yates v. GOSH, the Guardian and the United Kingdom: Reflections on the Legal Process and the Legal Principles’ (2017) 17 Med Law Int 285, 287.

24 M Brazier, ‘An Intractable Dispute: When Parents and Professionals Disagree’ (2005) 13 Med L Rev 412, 415.

25 There are some indications as to what the courts will consider to be the child’s best interests (or welfare, which the courts hold to be equivalent), although the principle remains underspecified. s 1(3) of the CA89 provides a list of factors that should be considered in determining the child’s welfare. The courts commonly determine medical decisions under the inherent jurisdiction and have asserted that ‘best interests’ go beyond medical interests to include interests related to emotion and other aspects of welfare. The courts have also determined a series of ‘intellectual milestones’ that should be considered in such cases. The decision must; be looked at from the child’s point of view; include a strong, but rebuttable, presumption in favour of preserving life; be made on the balance of all relevant factors, which may be drawn up in a balance sheet. Additionally, factors including quality of life, futility of treatment, and burdens and benefits of treatment have developed over time through the body of English case law that may also inform the discussion as to whether treatment is in a child’s best interest. See: *Re B (A Minor) (Wardship: Sterilisation)* HL [1988] AC 199, [206]; *Re A (Medical Treatment: Male Sterilisation)* [2000] 1 FCR 193; *Wyatt v Portsmouth NHS Trust* [2005] EWCA Civ 1181, [87]; consider also Huxtable (n 22).

26 Law Commission, Review of Child Law: Guardianship and Custody, No 172 (HMSO 1988): ‘… while the checklist may provide a clear statement of what society considers the most important factors in the welfare of children, it must not be applied too rigidly or be so formulated as to prevent the court from taking into account everything that is relevant in the particular case’ (3.19).

27 CM Lim, MC Dunn and JJ Chin, ‘Clarifying the Best Interests Standard: The Elaborative and Enumerative Strategies in Public Policy-making’ (2016) 42 J Med Ethics 542.; This liberal justification seems to post-date discussion of the CA89, although a similar justification of best interests based on informality is clearly visible in the 1991 Law Commission consultation preceding the introduction of the MCA: see Law Commission, Mentally Incapacitated Adults and Decision-Making: An Overview, No 119 (HMSO 1991) 4.14—our thanks to Lucy Series for offering this observation.

28
*Aintree University Hospitals NHS Foundation Trust v James* [2013] UKSC 67; *Wye Valley NHS Trust v B* [2015] EWCOP 60.

29 R Heywood, ‘Parents and Medical Professionals: Conflict, Cooperation, and Best Interests’ (2012) 20 Med Law Rev 29; C Auckland and I Goold, ‘Parental Rights, Best Interests and Significant Harms: Who Should Have the Final Say over a Child's Medical Care? (2019) 78(2) Camb Law J 287.

30
*Raqeeb* (n 8).

31 D Held, *Global Transformations: Politics, Economics and Culture* (Stanford UP 1999) 15.

32 ibid.

33 W Scheuerman, ‘Globalization’ in EN Zalta (ed), *The Stanford Encyclopedia of Philosophy* (Winter 2018 Edition) <https://plato.stanford.edu/archives/win2018/entries/globalization/> accessed 14 March 2020.

34 Some accounts of Globalisation (eg Held (n 31)) perceive the process of Globalisation as being as ancient as the history of humankind itself, with growth of contact between hitherto isolated societies forming a feedback loop with the emergence of trade and the transfer of successive technologies. The modern-era acceleration of these processes is highlighted by nineteenth and early twentieth-century commentators, including Karl Marx and John Dewey, who noted the revolutionary impact of technologies that reduced travelling and communication times on both the economics of the nation state and the finitude of human relationships. See K Marx and F Engels, *The Communist Manifesto* (Penguin Classics 2002) 13 and 14; J Dewey, *The Public and Its Problems: An Essay in Political Inquiry* (Swallow Press 2016) 117.

35 MB Steger, *Globalisms: The Great Ideological Struggle of the Twenty-first Century* (3rd edn, Lanham, Rowman & Littlefield Publishers 2008).

36 CH Tan and P Macneill, ‘Globalisation, Economics and Professionalism’ (2015) 37 Med Teach 850; J Frenk and O Gomez-Dantes, ‘Globalisation and the Challenges to Health Systems’ (2002) 325 BMJ 95.

37 The concentration of the much greater economic benefits to wealthy nations in the hands of tiny elites nevertheless means that many citizens in wealthy countries have also been losers due to greater globalisation. See J Stiglitz, *Globalization and Its Discontents Revisited: Anti-globalization in the Era of Trump* (Penguin Random House 2017).

38 Examples include off-shoring of medical trials by pharmaceutical companies, active targeting of jurisdictions with weaker consumer protections to promote activities harmful to human health such as tobacco smoking and mining. See A Petryna, *When Experiments Travel: Clinical Trials and the Global Search for Human Subjects* (Princeton UP 2009); RA Suliankatchi and others, ‘Smokeless Tobacco Use Is "Replacing" the Smoking Epidemic in the South-East Asia Region’ (2019) 21 Nicotine Tob Res 95; R Moody, *Rocks and Hard Places: The Globalisation of Mining* (Zed Books 2007).

39 B Lovelock, K Lovelock and K Lyons, ‘The Impact of Outbound Medical (Dental) Tourism on the Generating Region: New Zealand Dental Professionals’ Perspectives' (2018) 67 Tourism Management 399. Other beneficiaries include some healthcare professionals who can migrate to wealthier jurisdictions where they meet a demand for shortage skills, radically boosting their personal income and accessing better training and career opportunities see: IG Cohen, ‘Medical Tourism, Medical Migration, and Global Justice: Implications for Biosecurity in a Globalized World’ (2017) 25 Med Law Rev 200

40 See Harrington’s extended analysis of *N v Secretary of State for The Home Department* [2005] UKHL 31 in J Harrington, *Towards a Rhetoric of Medical Law* (Routledge 2017) 42–63.

41 Cohen (n 39).

42 Tan and Macneill (n 36); VA Crooks and others, ‘"Medical Tourism Will … Obligate Physicians to Elevate Their Level So That They Can Compete": A Qualitative Exploration of the Anticipated Impacts of Inbound Medical Tourism on Health Human Resources in Guatemala’ (2019) 17 Hum Resour Health 53.

43 IG Cohen, ‘Protecting Patients with Passports: Medical Tourism and the Patient-Protective Argument’ (2010) 95 Iowa L Rev 1467; These citizens are also the most affected by the growing commodification of human bodies, supplying burgeoning markets for international child surrogacy and organ trafficking, see Harrington (n 11); IG Cohen, ‘Circumvention Tourism’ (2012) 97 Cornell L Rev 1309.

44 M Castells, *Networks of Outrage and Hope* (2nd edn, Polity Press 2015)

45 Stiglitz (n 37).

46 McNamee (n 12); M Lynch, *Know-it-all Society: Truth and Arrogance in Political Culture* (Liveright 2019).

47 Harrington (n 11); Cohen (n 43).

48 T Ehrbeck, C Guevara and PD Mango, ‘Mapping the Market for Medical Travel’ (2008) 11 The McKinsey Quarterly 1; M Jaapar and others, ‘Dental Tourism: Examining Tourist Profiles, Motivation and Satisfaction’ (2017) 61 Tourism Management 538; J Shin and others, ‘Utilization Status and Satisfaction with Medical Services in Nonresidential Foreign Medical Tourists Visiting a Korean Medicine Hospital’ (2018) 2018 Evid Based Complement Alternat Med 6586352.

49 N Lunt, D Horsfall and J Hanefeld, ‘Medical Tourism: A Snapshot of Evidence on Treatment Abroad’ (2016) 88 Maturitas 37

50 VA Crooks and others, ‘What Is Known about the Patient’s Experience of Medical Tourism? A Scoping Review' (2010) 10 BMC Health Serv Res 266.

51 Lunt, Horsfall and Hanefeld (n 49); Lovelock, Lovelock and Lyons (n 39).

52 Tan and Macneill (n 36).

53 N Cortez, ‘Patients Without Borders: The Emerging Global Market for Patients and the Evolution of Modern Health Care’ (2008) 83 Indiana Law J 71; Cohen (n 43).

54 Lovelock, Lovelock and Lyons (n 39).

55 R Jeevan, J Birch and AP Armstrong, ‘Travelling Abroad for Aesthetic Surgery: Informing Healthcare Practitioners and Providers While Improving Patient Safety’ (2011) 64 J Plast Reconstr Aesthet Surg 143.

56 J Rossello, M Santana-Gallego and W Awan, ‘Infectious Disease Risk and International Tourism Demand’ (2017) 32 Health Policy Plan 538; SS Cai, K Chopra and SD Lifchez, ‘Management of Mycobacterium Abscessus Infection after Medical Tourism in Cosmetic Surgery and a Review of Literature’ (2016) 77 Ann Plast Surg 678; Cohen (n 39).

57 Consider commentary on the failure to observe lockdown precautions of one of the leading scientists advising the UK government on the COVID-19 pandemic used ad hominem attacks on hypocrisy to attack the science behind the precautions themselves, eg T Young, ‘Professor Lockdown’s Spell Has Been Broken’ (*The Spectator*, 9 May 2020) <https://www.spectator.co.uk/article/professor-lockdowns-spell-has-been-broken> accessed 2 August 2020; V Chalmers and L May, ‘Computer Code for Prof Lockdown's Model Which Predicted 500,000 Would Die from Covid-19 and Inspired Britain's “Stay Home” Plan Is a “Mess Which Would Get You Fired in Private Industry” Say Data Experts’ *Daily Mail* (17 May 2020) <https://www.dailymail.co.uk/news/article-8327641/Coronavirus-modelling-Professor-Neil-Ferguson-branded-mess-experts.html> accessed 2 August 2020.

58 For example, A Kamradt-Scott, ‘Explainer: What Donald Trump’s Funding Cuts to WHO Mean for the World’ (*The Conversation*, 15 April 2020) <https://theconversation.com/explainer-what-donald-trumps-funding-cuts-to-who-mean-for-the-world-136384>; ‘Coronavirus Lockdown Protest: What's Behind the US Demonstrations?’ (*BBC News*, 21 April 2020) <https://www.bbc.co.uk/news/world-us-canada-52359100>.

59 JL Dunoff, ‘Does Globalisation Advance Human Rights’ (1999) 25 Brook J Int'l L 125.

60 Cohen (n 39).

61 Harrington (n 11).

62 L Culley and others, ‘Children Travelling for Treatment: What We Don’t Know' (2013) 98 Arch Dis Child 442.

63 M Castells, *The Internet Galaxy: Reflections on the Internet, Business, and Society* (OUP 2001); Castells (n 44).

64
*An NHS Trust v SR* [2012] EWHC 3842 (Fam).

65 R Kisiel and E Sinmaz, ‘Seven-year-old Boy Whose Mother Ran Away with Him to Stop His Life-saving Cancer Treatment Is Taken Into Foster Care’ *Daily Mail* (6 December 2012). <https://www.dailymail.co.uk/news/article-2243542/Neon-Luca-Roberts-taken-foster-care-mother-tried-stop-life-saving-cancer-treatment.html> accessed 14 March 2020.

66 ‘AGAINST ALL ODDS: The Sally Roberts Story’ <https://sallyrobertsourstory.wordpress.com/sally-roberts-story/> accessed 14 March 2020.

67 Letter from Sally Roberts to Prime Minister David Cameron (24 January 2013) <http://joininghandsinhealth.com/public/Prime%20Minister_openlet_240113.pdf>.

68 ‘Appeal to Raise Money for Neon Roberts’ (*GoFundMe*, 14 January 2013) <https://www.gofundme.com/f/neonappeal> accessed 14 March 2020.

69
*King* (n 7).

70
*Great Ormond Street v Yates* (n 7).

71 ‘Charlie Gard #charliesfight’ (*GoFundMe*, 30 January 2017) <https://www.gofundme.com/f/please-help-to-save-charlies-life> accessed 15 March 2020.

72 D Connett, ‘Great Ormond Street Staff “Get Death Threats” over Charlie Gard’ *The Guardian* (23 July 2017) <https://www.theguardian.com/uk-news/2017/jul/22/great-ormond-street-staff-receive-death-threats-over-charlie-gard> accessed 16 March 2020.

73
*Kings v Thomas* (n 6).

74
*Evans* (n 8)

75 B Quinn and K Rawlinson, ‘Alfie Evans: Police Issue Warning over Online Abuse of Medical Staff’ *The Guardian* (26 April 2018) <https://www.theguardian.com/uk-news/2018/apr/25/alfie-evans-struggling-after-treatment-withdrawn-court-told> accessed 16 March 2020.

76
*Raqeeb* (n 8).

77 H Siddique, ‘Mother Seeks to Make Seriously Ill Girl Italian Citizen after Legal Win’ *The Guardian* (16 October 2019) <https://www.theguardian.com/society/2019/oct/16/mother-seeks-to-make-seriously-ill-girl-italian-citizen-after-legal-win-tafida-raqeeb> accessed 14 March 2020.

78
*Great Ormond Street Hospital v Yates* [2017] EWHC 1909 (Fam), [11].

79 ibid.

80 Auckland and Goold (n 29); F Nelson, ‘The Agony of Alfie Evans’ Parents Was Made Worse by Bad Law’ *The Spectator* (28 April 2018) <https://www.spectator.co.uk/article/the-agony-of-alfie-evans-parents-was-made-worse-by-bad-law> accessed 14 March 2020.

81 ‘NHS Trust Treating Brain-damaged Boy Gets Anonymity over Abuse Fears’ *The Guardian* (12 December 2017) <https://www.theguardian.com/society/2017/dec/11/nhs-trust-treating-brain-damaged-boy-gets-anonymity-over-abuse-fears> accessed 14 March 2020.

82 [2020] EWCA Civ 164.

83 ibid [102].

84 ibid.

85 Sir J Munby, ‘The Crisis in Private Law’ (*Transparency Project*, 10 February 2020) <http://www.transparencyproject.org.uk/the-crisis-in-private-law-by-sir-james-munby/> accessed 14 March 2020; O Bowcott, ‘Reporting Restrictions Turning Family Courts into Black Hole, Say Ex-judge’ *The Guardian* (11 February 2020) <https://www.theguardian.com/law/2020/feb/11/reporting-restrictions-family-courts-black-hole-judge> accessed 14 March 2020.

86 N Bhatia, ‘Three Ways the Charlie Gard Case Could Affect Future End-of-life Cases Globally’ (*The Conversation*, 25 July 2018) <https://theconversation.com/three-ways-the-charlie-gard-case-could-affect future-end-of-life-cases-globally-81168> accessed 14 March 2020.

87 R Das, ‘Populist Discourse on a British Social Media Patient-Support Community: The Case of the Charlie Gard Support Campaign on Facebook’ (2018) 24 Discourse, Context & Media 76.

88 Sean Parker, the first president of Facebook,reportedly explained at a public event that Facebook and other types of social media were consciously designed to exploit human psychology by rewarding user’s attention with a steady drip of social approbation. See M Allen, ‘Sean Parker Unloads on Facebook: “God Only Knows What It's Doing to Our Children's Brains”’ (*Axios*, 9 November 2017) <https://www.axios.com/sean-parker-unloads-on-facebook-2508036343.html> accessed 26 June 2020.

89 McNamee (n 12).

90 Lynch (n 46).

91 M Gabielkov and others, ‘Social Clicks: What and Who Gets Read on Twitter?’ (Proceedings of the 2016 ACM SIGMETRICS International Conference on Measurement and Modeling of Computer Science, June 2016) 179.

92 Lynch (n 46) citing MJ Crockett, ‘Moral Outrage in the Digital Age’ (2017) 1 Nat Hum Behav 769

93 Lynch, ibid 59.

94 Despite their deaths, Facebook groups remain active for Charlie Gard and Alfie Evans; however, the objectives of these groups have now changed to focus on law reform and advocacy for other parents with critically ill children.

95 Das (n 88).

96 These included: #charliesarmy; #charliesfight; #alfiesarmy; alfieswar; and #lifeforIsaiah.

97 The use of significant media and social media to attract global support is a novel phenomenon in high-profile cases in the UK paediatric context. It is somewhat presaged by the prominence of the media and public involvement in cases involving adults in the USA (Tery Schiavo) and Italy (Eluana Englaro).

98 Including the Pope, President Trump, and the President of Poland, Andrzej Duda.

99 G Hinsliff, ‘Alfie Evans’ Parents Needed Help. The Vultures Came Instead’ *The Guardian* (27 April 2018) <https://www.theguardian.com/commentisfree/2018/apr/26/alfie-evans-parents-activists> accessed 14 March 2020; R Hurley, ‘How a Fight for Charlie Gard Became a Fight Against the State’ (2017) 358 Br Med J j3675.

100 AG Klein, ‘How Online Hate Infiltrates Social Media and Politics’ (*The Conversation*, 16 March 2017) <https://theconversation.com/how-online-hate-infiltrates-social-media-and-politics-74353> accessed 14 March 2020.

101 This was particularly illuminated in the *Gard* and *Evans* cases. See J Street-Porter, ‘I Know How Charlie Gard’s Parents Feel – But Their Trauma Shouldn’t Be Exploited for Political Gain’ *The Independent* (7 July 2017) <https://www.independent.co.uk/voices/charlie-gard-gosh-great-ormond-street-hospital-ruling-pope-donald-trump-a7829351.html> accessed 14 March 2020.

102 A Gallagher, ‘What Can We Learn from the Case of Charlie Gard? Perspectives from an Inter-disciplinary Panel Discussion’ (2017) 24 Nurs Ethics 775, 775

103 Hurley (n 100).

104 This is also being seen in broader society, where a range of social issues, such as climate change, and vaccinations for children have been the focal point media commentators and campaigns based on scepticism, or outright denial, of mainstream science. See, for example, NT Gavin and T Marshall, ‘Mediated Climate Change in Britain: Scepticism on the Web and on Television Around Copenhagen’ (2011) 21 Global Environ Chang 3 1035; C Booker, ‘The Climate Change Brigade Are Wrong Again’ *Daily Telegraph* (13 August 2016) <https://www.telegraph.co.uk/news/2016/08/13/the-climate-change-brigade-are-wrong-again/> accessed 14 March 2020.

105 TM Paulusa and KR Roberts, ‘Crowdfunding a “Real-life Superhero”: The Construction of Worthy Bodies in Medical Campaign Narratives’ (2018) 21 Discourse, Context & Media 64.

106 MJ Young and E Scheinberg, ‘The Rise of Crowdfunding for Medical Care: Promises and Perils’ (2017) 317 J Am Med Assoc 1623. Other types of crowdfunding include: reward based crowdfunding; equity crowdfunding; and debt crowdfunding.

107 Internet use in Britain was about two-thirds of today’s levels by the time of the case in 2012, and Neon’s mother’s letter shows that research into novel treatment alternatives was an option for parents at this time. Social media use was prevalent in 2012, with more than 50% of adults in England using Facebook. Yet this is far below current levels—77% in the UK in one survey. see J Johnson, ‘Daily Internet Users in Great Britain 2006-2019’ (*Statista*, 14 February 2020) <https://www.statista.com/statistics/275786/daily-internet-users-in-great-britain/>; Department of Culture, Media and Sport, ‘Taking Part: Focus on Social Media’ (*Department of Culture, Media and Sport*, April 2016) <https://assets.publishing.service.gov.uk/government/uploads/system/uploads/attachment_data/file/519678/Social_media_-_FINAL.pdf> accessed 20 June 2020; UMPF, ‘Social Media Usage in the UK – The Findings’ (*UMPF*, 2019) <https://umpf.co.uk/social-media-usage-in-the-uk-the-findings/> accessed 20 June 2020.

108 See M Cikara and JJ Van Bavel, ‘The Neuroscience of Intergroup Relations: An Integrative Review’ (2014) 9(3) Perspect Psychol Sci 245; DA Vaughn and others, ‘Empathic Neural Responses Predict Group Allegiance’ (2018) 12 Front Hum Neurosci 302.

109 See n 72. Conversely, crowdfunding has been used by fringe groups to raise funds for their causes, and recently popular crowdfunding sites have banned some of these groups from accessing their sites. See B Montgomery, ‘PayPal, GoFundMe, and Patreon Banned a Bunch of People Associated with the Alt-Right. Here’s Why’ *Buzzfeed News* (2 August 2017) <https://www.buzzfeednews.com/article/blakemontgomery/the-alt-right-has-a-payment-processor-problem> accessed 14 March 2020.

110 G Dressler and SA Kelly, ‘Ethical Implications of Medical Crowdfunding: The Case of Charlie Gard’ (2018) 44 J Med Ethics 453.

111 The term ‘sharenting’ is relevant here. It refers to the practice of parents oversharing information related to their children on social media. This may include photographs or videos detailing their child’s daily lives on platforms such as Facebook, Twitter, or Instagram. See E Nottingham, ‘Children’s Rights to Privacy in the Age of “Generation Tagged”: Sharenting, Digital Kidnapping and the Child Micro-celebrity’ in Jane Murray, Beth Blue Swadener and Kylie Smith (eds), *The Routledge International Handbook of Young Children's Rights* (Routledge, London 2019) 16.

112 S Kale, ‘When Survival Is a Popularity Contest: The Heartbreak of Crowdfunding Healthcare’ *The Guardian* (20 May 2019) <https://www.theguardian.com/lifeandstyle/2019/may/20/when-survival-is-a-popularity-contest-the-heartbreak-of-crowdfunding-healthcare> accessed 14 March 2020.

113 ibid 455.

114
*NHS v SR* (n 65).

115
*R v Cambridge Health Authority, ex parte B* [1995] 2 All ER 129; *Simms v Simms; A v A (a child)* [2002] EWHC 2734.

116 G Birchley and others, ‘Conceptualising Surgical Innovation: An Eliminativist Proposal’ (2020) 28(1) Health Care Anal 73.

117
*Simms* (n 116). Simms concerned an 18- and 16-year-old, JS and JA, both in the advanced stages of a fatal neurodegenerative disease. The parents sought a declaration that they could have an experimental treatment, never tested on humans that involved infusing a substance directly into the brain. The hospital trust and the official solicitor opposed the application. While animal tests in a similar disease were promising, the effects of treating humans were unpredictable, and administering treatment risked potentially catastrophic mishaps. Nevertheless, the court was swayed by testimony from the families and nursing teams that the children had sufficient quality of life to make treatment extending their survival worthwhile. Although JA defied medical expectations and survived for a decade after treatment, ultimately the treatment did not prove efficacious. See: ‘Belfast Man with vCJD Dies after Long Battle’ (*BBC News*, 7 March 2011) <https://www.bbc.com/news/uk-northern-ireland-12667709> accessed 15 March 2020; on reasons for the ultimate inefficacy of the treatment, see ‘The Rise and Fall of Pentosan Polysulfate in Prion Disease’ (*CureFFI.org*, 13 December 2012 <www.cureffi.org/2012/12/13/the-rise-and-fall-of-pentosan-polysulfate-in-prion-disease/> accessed 15 March 2020.

118 C Garone and others, ‘Deoxypyrimidine Monophosphate Bypass Therapy for Thymidine Kinase 2 Deficiency’ (2014) 6(8) EMBO Mol Med 1016; C Lopez-Gomez and others, ‘Deoxycytidine and Deoxythymidine Treatment for Thymidine Kinase 2 Deficiency’ (2017) 81(5) Ann Neurol 641.

119 The trial included 18 children: 13 increased their body mass index, and one patient whose walking ability had improved over a 4-year spell.

120 See E Cave and E Nottingham, ‘Who Knows Best (Interests)? The Case of Charlie Gard’ (2017) 26 Med Law Rev 500.

121 LF Dai and others, ‘Phenotype and Genotype of Twelve Chinese Children with Mitochondrial DNA Depletion Syndromes’ (2019) 57(3) Chinese Journal of Pediatrics 211.

122 In this case, photodynamic therapy, which remains experimental in children, with few reported cases of use. See M Schwake and others, ‘In-vitro Use of 5-ALA for Photodynamic Therapy in Pediatric Brain Tumors’ (2018) 83 Neurosurgery 1328. Further note that photodynamic therapy has a range of less novel applications (including treatment) in other settings, eg BJ Qumseya, W David and HC Wolfsen, ‘Photodynamic Therapy for Barrett’s Esophagus and Esophageal Carcinoma' (2013) 46 Clin Endosc 30.

123 Specifically Immunotherapy, which is sometimes used in conjunction with, rather than as a replacement for, conventional therapy. See: EJ Sayour and DA Mitchell, ‘Immunotherapy for Pediatric Brain Tumors’ (2017) 7 Brain Sci 137.

124 The other treatment in this category is hyperbaric oxygen therapy, which has some evidence of safety but none of efficacy as a treatment in Neon’s case. See: Y Aghajan and others, ‘Use of Hyperbaric Oxygen Therapy in Pediatric Neuro-oncology: A Single Institutional Experience’ (2019) 141 J Neurooncol 151.

125 J Adams, ‘Ashya King Cleared of Cancer Three Years after His Parents Abducted Him from Hospital for Treatment Abroad’ *The Telegraph* (3 March 2018) <https://www.telegraph.co.uk/news/2018/03/03/ashya-king-cleared-cancer-three-years-parents-abducted-hospital/> accessed 14 March 2020.

126 T Yock and others, ‘Long-term Toxic Effects of Proton Radiotherapy for Paediatric Medulloblastoma: A Phase 2 Single-arm Study’ (2016) 17 Lancet Oncol 287.

127 ‘Proton Beam Therapy “Effective” and “causes Fewer Side Effects’” (*NHS*, 1 February 2016) <https://www.nhs.uk/news/cancer/proton-beam-therapy-effective-and-causes-fewer-side-effects/> accessed 14 March 2020.

128 G Mezzofiore, ‘So Ashya's Parents Were RIGHT: Proton Beam Cancer Therapy That Forced Family to Go on the Run to Spain Because They Couldn't Get It on the NHS Is As Good As Chemotherapy - And Has Fewer Side Effects’ *Mail Online* (30 January 2016) <https://www.dailymail.co.uk/news/article-3424058/So-Ashya-s-parents-RIGHT-Proton-beam-cancer-therapy-forced-family-run-Spain-couldn-t-NHS-good-chemotherapy-fewer-effects.html> accessed 14 March 2020.

129 ‘Proton Beam Therapy’ (*NHS The Christie Foundation Trust*) <http://www.christie.nhs.uk/our-future/our- developments/protons/proton-beam-therapy.aspx;> accessed 14 March 2020.

130 NHS England, ‘Clinical Commissioning Policy: Proton Beam Therapy for Children, Teenagers and Young Adults in the Treatment of Malignant and Non-malignant Tumours’ (2018) <https://www.england.nhs.uk/commissioning/wp-content/uploads/sites/12/2019/07/Interim-Policy-PBT-for-CTYA-for-malignant-and-non-malignant-tumours.pdf> accessed 23 June 2020.

131 M Hynuh and others, ‘Are Further Studies Needed to Justify the Use of Proton Therapy for Paediatric Cancers of the Central Nervous System? A Review of Current Evidence’ (2019) 133 Radiother Oncol 140.

132 M Durante, ‘Proton Beam Therapy in Europe: More Centres Need More Research’ (2019) 120 Br J Cancer 777.

133
*R v Cambridge* (n 116).

134 ibid 133.

135
*Evans* (n 8) [43].

136 Concerns have also be raised about the wider impacts on families, see FA Carnevale and others, ‘Daily Living with Distress and Enrichment: The Moral Experience of Families with Ventilator-assisted Children at Home’ (2006) 117 Pediatrics 48.

137 D Wilkinson and L Gillam, ‘Should Long-term Ventilation Be Offered in Severe Spinal Muscular Atrophy’ (2013) 49 J Paediatr Child Health 813; WE Novotny and others, ‘Mechanical Ventilation for a Child with Quadriplegia’ (2014) 134 Pediatrics 593

138 M Fine-Goulden, S Ray and J Brierley, ‘Decision-making in Long-term Ventilation for Children’ (2015) 3 Lancet Respir Med 745

139 D Wilkinson and J Savulescu, *Ethics, Conflict and Medical Treatment for Children: From Disagreement to Dissensus* (Elsevier 2019); D Wilkinson, ‘Debate Response: Charlie Gard, Interests and Justice – An Alternative View’ (*Journal of Medical Ethics Blog*, 26 April 2017) <https://blogs.bmj.com/medical-ethics/2017/04/26/debate-reponse-charlie-gard-interests-and-justice-an-alternative-view/> accessed 14 March 2020.

140 J Lantos, ‘The Tragic Case of Charlie Gard’ (2017) 171 JAMA Pediat 935.

141 RD Truog, ‘The United Kingdom Sets Limits on Experimental Treatments: The Case of Charlie Gard’ (2017) 318 JAMA 318.

142 G Birchley, ‘The Harm Threshold: A View from the Clinic’ in I Goold, J Herring, C Auckland (eds), *Parental Rights, Best Interests and Significant Harms: Medical Decision-Making on Behalf of Children Post-Great Ormond Street Hospital v Gard* (Hart 2019).

143 JB McKinlay, ‘From Promising Report to Standard Procedure - 7 Stages in the Career of a Medical Innovation’ (1981) 59(3) Milbank Q 374.

144 K Hutchison, J Johnson and D Carter, ‘Justice and Surgical Innovation: The Case of Robotic Prostatectomy’ (2016) 30(7) Bioethics 536.

145 Dr B Port, ‘Trials to Treatments: Gene Therapy’ (*Parkinson’s UK*, 11 June 2018) <https://medium.com/parkinsons-uk/trials-to-treatments-gene-therapy-143e6a1b4a24> accessed 14 March 2020.

146 CS Broberg and others, ‘Emergence of the Arterial Switch Procedure for Transposition of the Great Arteries and the Potential Cost of Surgical Innovation’ (2017) 154 J Thorac Cardiovasc Surg 1047.

147 K Wright, ‘Making Unbearable Decisions about the Care and Treatment of a Seriously Ill Child – Ethical Reflections’ (*Nuffield Council on Bioethics*, 7 August 2017) <http://nuffieldbioethics.org/blog/making-unbearable-decisions-care- treatment-ill-child> accessed 14 March 2020.

148 A Rothstein and A Lewis, ‘The Challenges of Discussing “Longshot” and “Fantasy” Treatments’ (2018) 18 Am J Bioeth 27.

149 Consider, for example, the father’s testimony at [20] in *Re X (A Child)* [2015] EWHC 2778 (Fam), or the mother’s interpretation of events in *NHS v BK* (n 9).

150
*NHS v BK*, ibid [69].

151
*NHS v SR* (n 65) [25].

152
*B v D and MOD* [2017] EWCOP 15.

153 C Hamlyn-Williams, M Lakhanpaul and L Manikam, ‘Child Medical Tourism: A New Phenomenon’ in N Lunt, D Horsfall and J Hanefeld (eds), *Handbook on Medical Tourism and Patient Mobility* (Edward Elgar Publishing 2015). Several academics have argued that the term ‘medical tourism’ is crass, and that the term ‘tourist’ is often associated with enjoyment and pleasure, and that those who travel for medical treatment often do so due to pain, desperation, or suffering hardship having to travel for life-saving treatment. See D Horsfall, ‘Medical Tourism from the UK to Poland: How the Market Masks Migration’ (2019) J Ethn Migr Stud 1469, doi: 10.1080/1369183X.2019.1597470; J Connell, ‘Medical Tourism - Concepts and Definitions’ in Neil Lunt, Daniel Horsfall and Johanna Hanefeld (eds), *Handbook on Medical Tourism and Patient Mobility* (Edward Elgar Publishing 2015). We note that tourism is defined by the The World Tourism Organization as ‘compris[ing] the activities of persons travelling to and staying in places outside their usual environment for not more than one consecutive year for leisure, business and other purposes’, and health tourism as ‘Tourism associated with travel to health spas or resort destinations where the primary purpose is to improve the traveller's physical well-being through a regimen of physical exercise and therapy, dietary control, and medical services relevant to health maintenance.’ See Y Gee and E Fayos-Solá, *International Tourism: A Global Perspective* (World Tourism Organization 1997).

154 Letter from Sally Roberts to Prime Minister David Cameron (n 68).

155
*Re JM (A Child)* [2015] EWHC 2832 (Fam).

156 Mostyn J thought that parents preferred Chinese Medicine, although it is unclear what, if any, treatment JM received given his disappearance.

157
*King* (n 7) [33] (Justice Baker).

158 ‘Alfie Evans Granted Italian citizenship in Life Support Legal Row’ (*BBC News*, 23 April 2018) <https://www.bbc.com/news/uk-england-merseyside-43872678> accessed 14 March 2020.

159
*Raqeeb* (n 8) [155].

160 Siddique (n 78).

161
*R v Cambridge* (n 114).

162 Hamlyn-Williams, Lakhanpaul and Manikam (n 154).

163 Culley and others (n 62).

164 These phenomena have been described in adult members of ethnic minority communities. See N Lunt, ‘The United Kingdom’s Somali Populations as Medical Nomads’ (2019) J Ethn Migr Stud 1, doi: 10.1080/1369183X.2019.1597466.

165 See Hayley Dixon, ‘Terminally Ill Boy Denied “Potentially Life-saving” Treatment by NHS “Would Be Given It in Any US Hospital”’ *The Telegraph* (3 April 2017) <https://www.telegraph.co.uk/news/2017/04/03/terminally-boy-denied-potentially-life-saving-treatment-nhs/> accessed 14 March 2020.

166 ‘Corey Ashcroft Returns Home after Cancer Treatment’ (*ITV News*, 18 July 2014) <https://www.itv.com/news/granada/2012-04-13/corey-ashcroft-returns-home-after-cancer-treatment/> accessed 14 March 2020.

167 Wilkinson and Savulescu (n 140).

168
*Re N* [2015] EWCA Civ 1112.

169 J Munby, ‘A Matter for the Parents? A Matter for the Judge? Thoughts on 30 Years of the Children Act and the Revival of the Inherent Jurisdiction’ (2019) Family Law (March) 264.

170 Birchley (n 143).

171
*Great Ormond Street Hospita* (n 7) [90] (Francis J).

172 J Lee and F Lynn, ‘Mental Health and Well-being of Parents Caring for a Ventilator-dependent Child’ (2017) 29 Nurs Child Young People 33; V Zamvar, G Lazonby and JW Puntis, ‘Recurrent Life-threatening Sepsis in Intestinal Failure: Transplantation or Foster Care?’ (2013) 98 Arch Dis Child 556.

173 B Lindahl and BM Lindblad, ‘Family Members' Experiences of Everyday Life When a Child Is Dependent on a Ventilator: A Metasynthesis Study’ (2011) 17 J Fam Nurs 241, 263.

174 Lee and Lynn (n 173).

175 J Hanefeld and others, ‘What Do We Know about Medical Tourism? A Review of the Literature with Discussion of Its Implications for the UK National Health Service as an Example of a Public Health Care System’ (2014) 21(6) J Travel Med 410

176 D Horsfall and N Lunt, ‘Medical Tourism by Numbers’ in Lunt, Horsfall and Hanefeld (n 154).

177 Culley and others (n 62); K Adams, J Synder and VA Crooks, ‘The Perfect Storm: What’s Pushing Canadians Abroad for Dental Care?’ (2017) 83 J Can Dent Assoc 10.

178 VA Crooks and others, ‘"You Don’t Want to Lose That Trust That You've Built with This Patient …": (Dis)trust, Medical Tourism, and the Canadian Family Physician-Patient Relationship' (2015) 16 BMC Fam Pract 25; B Nelson, ‘A Trip to Remember, with Strings Attached: Researchers Begin Sorting Through the Tangled Ethical and Legal Implications of Medical Tourism’ (2014) 122 Cancer Cytopathol 787; VA Crooks and others, ‘Ethical and Legal Implications of the Risks of Medical Tourism for Patients: A Qualitative Study of Canadian Health and Safety Representatives’ Perspectives' (2013) 3 BMJ Open e002302.

179 Z Meghani, ‘A Robust, Particularist Ethical Assessment of Medical Tourism’ (2011) 11 Dev World Bioeth 16.

180 Lovelock, Lovelock and Lyons (n 39).

181 Cohen (n 43). According to Cohen, the most common types of treatments that are sought abroad in these types of cases are abortion, fertility treatments, surrogacy, organ transplantation, and medically assisted suicide for those with terminal illness.

182 M Ormond, ‘International Medical Travel, or Medical Tourism’ in A Kobayashi (ed), *International Encyclopedia of Human Geography* (2nd edn, Elsevier 2020) 373.

183 For example, on social media consider: Nottingham (n 112); McNamee (n 12), especially 291–315. On innovative treatment, consider V Larcher, H Turnham and J Brierley, ‘Medical Innovation in a Children's Hospital: “Diseases Desperate Grown by Desperate Appliance Are Relieved, or Not at All”’ (2018) 32(1) Bioethics 36.

184 S Jodoin and C Pollack, ‘Children’s Rights, International Trade Law and Economic Globalisation’ in C Fenton-Glynn (ed), *Children's Rights and Sustainable Development* (CUP 2019) 261.

185 Dunoff (n 59).

186 OJ Otusanya, ‘Corruption as an Obstacle to Development in Developing Countries: A Review of the Literature’ (2011) 14 JMLC 387.

187 Stiglitz (n 37).

188 J Tobin, ‘Justifying Children’s Rights' (2013) 21 Int J Child Rights 395.

189 arts 18 and 27.

190 Cohen (n 43).

191
*Re JM* (n 156).

192 We observe that in cancer treatment Traditional Chinese Medicine is almost invariably used in conjunction with Western medicine, rather than as a replacement, as it potentially was the case in *JM.* Nevertheless we acknowledge that there is considerable debate about the efficacy of Traditional Chinese Medicine in general, and our description of its use as a treatment in JM as suboptimal does not imply a position on this wider debate. See TH So and others, ‘Chinese Medicine in Cancer Treatment - How Is It Practised in the East and the West?’ (2019) 31 Clin Oncol (R Coll Radiol) 578.

193 [2017] EWCA 410, [115].

194 See, eg Auckland and Goold (n 29).

195 NK Sim, ‘The Travelling Patient: A Clinician’s Guide to the Law Surrounding Medical Tourism' (2014) 82 Med Leg J 159.

196
*Re K (A Child: Stranding: Forum Conveniens: Anti-Suit Injunction)* [2019] EWHC 466.

197 Sim (n 196).

198 [2011] EWHC 3298.

199 R Argawal, ‘Medical Malpractice’ (2015) 2 Court Uncourt 17.

200 K Amirthalingam, ‘Medical Negligence and Patient Autonomy - Bolam Rules in Singapore and Malaysia - Revisited’ (2015) 27 Sing Ac LJ 666; Sim (n 196).

201 Sim, ibid.

202 DJB Svantesson, ‘From the Airport to the Surgery to the Courtroom – Private International Law and Medical Tourism’ (2008) 34 Commonw Law Bull 265.

203 Sim (n 196).

204 N Cortez, ‘Embracing the New Geography of Health Care’: A Novel Way to Cover Those Left out of Health Reform' (2011) 84 S Cal L Rev 859.

205 Cortez (n 53).

206 A Podlaski, ‘Toward an International Constitution of Patient Rights’ (2016) 23 Ind J Global Legal Stud 893; For discussion of the reasons for this regulatory ‘race for the bottom’, and arguments that it is the result of political choice rather than an inevitable consequence of globalisation, see Stiglitz (n 37) 27–29.

207 Podlaski, ibid.

208 Even if these great obstacles were overcome, the history of existing international conventions suggests that they take many years to be agreed. One has only to examine the travails of the UN Convention on the Rights of Persons with Disabilities, which was first discussed in 1987 and not adopted until 2006. Furthermore, the current overt emphasis on national self-interest seen among some countries, most obviously US President Trump’s ‘America First’ rhetoric, clearly makes for a poor environment in which to forge fair international agreements.

209 Cortez (n 205); Cohen (n 39).

210 ibid; M Farid and others, ‘Complications of Cosmetic Surgery Abroad - Cost Analysis and Patient Perception’ (2019) 7 Plast Reconstr Surg Glob Open e2281.

211 J Hanefeld and others, ‘Medical Tourism: A Cost or Benefit to the NHS?’ (2013) 8 PLoS One e70406.

212 Sim (n 195).

213
*Raqeeb* (n 8) [29].

214 ‘Tafida Raqeeb: Brain-damaged Girl in High Court Case Out of Intensive Care’ (*BBC News*, 10 January 2020) <https://www.bbc.com/news/uk-england-london-51055153> accessed 15 March 2020.

215 S Griffiths, ‘Tafida Raqeeb: Parents of Girl on Life Support Call for Change in the Law’ *The Sunday Times* (8 March 2020) <https://www.thetimes.co.uk/article/our-tafida-5-is-alive-all-must-get-chance-to-keep-life-support-f2ttg9z6h> accessed 15 March 2020.

216 E Cave, J Brierley and D Archard, ‘Making Decisions for Children-Accommodating Parental Choice in Best Interests Determinations: Barts Health NHS Trust v Raqeeb [2019] EWHC 2530 (Fam); Raqeeb and Barts Health NHS Trust [2019] EWHC 2531 (Admin)’ (2020) 28(1) Med Law Rev 183.

217 K Donelson, ‘Medical Repatriation: The Dangerous Intersection of Health Care Law and Immigration’ (2015) 18 J Health Care L & Pol'y 347; Harrington (n 40).

218
*R v Cambridge* (n 116).

219 Notwithstanding some constrained discussion in *Gard* about this important issue: Therein Francis J stated ‘[the parents] have very publicly raised funds. What parents would not do the same? But I have to say, having heard the evidence that this case has never been about affordability, but about whether there is anything to be done for Charlie.’ *Great Ormond Street* (n 7) [16].

220 Robert J Holton, *Globalization and the Nation State* (Macmillan Education 2011).

221 Harrington (n 11).

## Notes

This article stems from a review conducted for the Nuffield Council on Bioethics entitled ‘Disagreements in the care of critically ill children: emerging issues in a changing landscape’ in September 2018. See https://www.nuffieldbioethics.org/publications/disagreements-in-the-care-of-critically-ill-children/literature-reviews. Giles Birchley’s work on this paper was part of the Balancing Best Interests in Medical Ethics and Law project (BABEL) generously funded by the Wellcome Trust (Grant Number 209841/Z/17/Z).

